# Fecal Microbiome Data Distinguish Liver Recipients With Normal and Abnormal Liver Function From Healthy Controls

**DOI:** 10.3389/fmicb.2019.01518

**Published:** 2019-07-03

**Authors:** Hai-Feng Lu, Zhi-Gang Ren, Ang Li, Hua Zhang, Shao-Yan Xu, Jian-Wen Jiang, Lin Zhou, Qi Ling, Bao-Hong Wang, Guang-Ying Cui, Xin-Hua Chen, Shu-Sen Zheng, Lan-Juan Li

**Affiliations:** ^1^State Key Laboratory for Diagnosis and Treatment of Infectious Disease, Collaborative Innovation Center for Diagnosis and Treatment of Infectious Diseases, The First Affiliated Hospital, School of Medicine, Zhejiang University, Hangzhou, China; ^2^Department of Infectious Diseases, Precision Medicine Center, The First Affiliated Hospital of Zhengzhou University, Zhengzhou, China; ^3^Department of Hepatobiliary and Pancreatic Surgery, The First Affiliated Hospital, School of Medicine, Zhejiang University, Hangzhou, China; ^4^Key Laboratory of Combined Multi-Organ Transplantation, Ministry of Public Health, Hangzhou, China; ^5^Health Management Center, The First Affiliated Hospital, School of Medicine, Zhejiang University, Hangzhou, China

**Keywords:** liver transplantation, intestinal microbiota, 16S rRNA gene sequencing, butyrate-producing bacteria, opportunistic pathogens

## Abstract

Emerging evidence suggests that altered intestinal microbiota plays an important role in the pathogenesis of many liver diseases, mainly by promoting inflammation via the “intestinal microbiota-immunity-liver” axis. We aimed to investigate the fecal microbiome of liver recipients with abnormal/normal liver function using 16S rRNA gene sequencing. Fecal samples were collected from 90 liver recipients [42 with abnormal liver function (Group LT_A) and 48 with normal liver function (Group LT_N)] and 61 age- and gender-matched healthy controls (HCs). Fecal microbiomes were analyzed for comparative composition, diversity, and richness of microbial communities. Principal coordinates analysis successfully distinguished the fecal microbiomes of recipients in Group LT_A from healthy subjects, with the significant decrease of fecal microbiome diversity in recipients in Group LT_A. Other than a higher relative abundance of opportunistic pathogens such as *Klebsiella* and *Escherichia*/*Shigella* in all liver recipients, the main difference in gut microbiome composition between liver recipients and HC was the lower relative abundance of beneficial butyrate-producing bacteria in the recipients. Importantly, we established a fecal microbiome index (specific alterations in *Staphylococcus* and *Prevotella*) that could be used to distinguish Group LT_A from Group LT_N, with an area under the receiver operating characteristic curve value of 0.801 and sensitivity and specificity values of 0.771 and 0.786, respectively. These findings revealed unique gut microbial characteristics of liver recipients with abnormal and normal liver functions, and identified fecal microbial risk indicators of abnormal liver function in liver recipients.

## Introduction

Liver transplantation is a common and effective therapy for end-stage liver diseases. As of 2015, more than 1,700 living-donor liver transplantations had been successfully performed at the First Affiliated Hospital, College of Medicine of Zhejiang University, China, with an overall 3 years post-surgery survival rate of more than 70%. Despite advances in low-toxicity immunosuppressive drugs, the long-term success of liver transplantation is still limited by the development of chronic liver allograft dysfunction (Starzl et al., 1985). Many studies have emphasized improving the liver transplantation surgery procedure and instituting personalized management of post-transplant patients, including minimization strategies for transplant immunosuppression, to decrease the development of unpredictable clinical complications such as acute and chronic rejection (Reyes et al., 2000), *de novo* autoimmunity (Fedoseyeva et al., 1999), fibrosing cholestatic hepatitis (Hori et al., 2016), infections, and chronic dysfunction (Kok et al., 2017). Additionally, underlying chronic illnesses such as hypertension, diabetes, dyslipidemia, and graft impairment all have severe impacts on the recovery of liver function, thus affecting longevity and quality of life of post-transplant patients (Schoening et al., 2013).

The human intestinal tract harbors about 100 trillion microbes. They play a role in the development of mucosal and systemic immunity and are also involved in host liver disease (Lu et al., 2011; Qin et al., 2014; Chen et al., 2016). Intestinal dysbiosis not only disturbs intestinal immune homeostasis but can also cause immune dysfunction of other non-enteric organs (Jiang et al., 2011; Smolinska and O’Mahony, 2016; Zeevi et al., 2016), which is associated with the induction and progression of liver damage (Garcia-Tsao and Wiest, 2004; Ren et al., 2013). Although liver disease is not necessarily a consequence of these disruptions, diseases of the liver always result in downstream intestinal microbiome and immune dysbiosis (Qin et al., 2014; Lv et al., 2016). Malnutrition, ischemia-reperfusion injury (Ren et al., 2013), and immunosuppression therapy in liver transplant (LT) recipients directly lead to dysbiosis, disrupted intestinal barriers, and alterations in the innate immune response. Importantly, most LT patients have had severe liver disease and complications for a long period prior to receiving a transplant, and are subject to immunosuppression (Fenkel and Halegoua-DeMarzio, 2016) and antibiotic therapy after transplantation (Lu et al., 2013). Although immunosuppressors (e.g., FK506 and CsA) were initially reported to impair intestinal permeability in animals (Gabe et al., 1998), clinical studies on the chronic effects of FK506 and CsA on the intestine of liver recipients 2–4 years post-orthotopic LT (Parrilli et al., 2003) showed that these immunosuppressors did not affect intestinal permeability.

An understanding of variations in the intestinal microbial profile of LT recipients is critical for appropriately managing these patients in the early post-LT period. This knowledge will allow calculated modulation of intestinal microbiome components to benefit restoration of the intestinal microbiota and the recovery of liver function soon after LT, thus improving quality of life and long-term survival. Therefore, in this study we examined the fecal microbiomes of LT recipients (Group LT, 24 months < post-LT period < 48 months) and HCs (Group HC) to uncover the characteristics of the repopulated intestinal microbiota of LT recipients within the stable post-LT period in the absence of any microbiome-targeted therapeutic interventions.

## Materials and Methods

### Study Design and Enrolled Patients

This study was approved by the Institutional Review Board of the First Affiliated Hospital, School of Medicine, Zhejiang University (IRB no. 2014-336). All experiments were performed in accordance with the Helsinki Declaration and the Rules of Good Clinical Practice, and no organs from executed prisoners were used in these recipients. Patients were considered for enrollment if they diagnosed with pathologically-diagnosed hepatitis B virus (HBV)-associated hepatocellular carcinoma with the Barcelona Clinic Liver Cancel (BCLC) stage A or B, and the Child -Pugh class A or B who met the Hangzhou criteria and had undergone liver transplantation more than 24 months but less than 48 months prior to the study period. Each participant filled out a baseline questionnaire (Supplementary Table S1). The inclusion criteria were: (a) aged 35–65 years with normal body weight, (b) diagnosed with hepatocellular carcinoma prior to LT and without microbial infection during the perioperative period (based on the patient’s medical history), (c) no obvious bodily discomfort, (d) no antibiotic use in the 12 weeks prior to enrollment and no probiotics and/or prebiotics after LT, and (e) FK506 tacrolimus used as the sole immunosuppressive therapy for more than 12 months. The exclusion criteria for patients were: (a) presence of severe complications such as liver abscess, recurrence of hepatocellular carcinoma (HCC), liver trauma, diabetes, hypertension, biliary and/or vascular complications through measuring serum CRP, PCT, and imaging examination, etc. (b) infection with human immunodeficiency virus, hepatitis C virus, or other types of hepatitis virus except HBV, (c) presence of any other organ-specific diseases, including intestinal diseases, pancreatic diseases, and/or tumor recurrence, (d) consumption of alcohol, tobacco, Chinese herbal medicine, and/or recreational drugs, and (e) staying up late, work fatigue and so on. Fecal samples and patient information (including data on diet, drug use, and alcohol consumption) were collected during periodic outpatient follow-up appointments between 1 December, 2014, and 31 December, 2016, at one of the two participating hospitals (First Affiliated Hospital School of Medicine, Zhejiang University, and Shulan (Hangzhou) Hospital). HCs (Group HC) matched to the age, gender, and BMI of the LT group were correspondingly screened and enrolled according to the inclusion and exclusion criteria described in our previous study (Lu et al., 2011). This study included a total of 151 fecal samples (90 from LT patients and 61 from HC) from the participants described above. Written informed consent and questionnaires addressing previous and current diseases, lifestyles, and medications were obtained from all subjects. The data of personalized perioperative medication management and professional postoperative care were extracted from electronic medical records.

### Sample Collection and DNA Extraction

Fecal samples were collected in sterile bags, refrigerated, and then taken directly to the laboratory. The samples were then divided into 200 mg aliquots, frozen rapidly in liquid nitrogen, and stored at −80°C until use.

Phenol trichloromethane was used to extract DNA from each frozen fecal sample aliquot using a bead beater to mechanically disrupt the cells, followed by phenol-chloroform extraction (Qin et al., 2014). Extracted DNA was quantified using a Qubit 2.0 Fluorometer (Invitrogen, Carlsbad, CA, United States), and molecular sizes were estimated using agarose gel electrophoresis. All fecal microbial DNA samples were diluted to a concentration of 10 ng/μl for microbial analysis.

### PCR and Sequencing

16S rRNA gene sequences were amplified from each of the extracted DNA samples using a set of primers targeting the hyper-variable V3-V4 region (338F/806R) of the gene: 338F, 5′-barcode-ACTCCTACGGGAGGCAGCA-3′ and 806R, 5′-GGACTACHVGGGTWTCTAAT-3′. PCR amplification was performed as described in our previous study (Lu et al., 2016). DNA libraries were constructed using kits provided by Illumina Inc. according to the manufacturer’s instructions, and DNA sequencing was performed using the Illumina MiSeq 2000 platform (San Diego, CA, United States) at the State Key Laboratory for Diagnosis and Treatment of Infectious Diseases (Zhejiang University, Hangzhou, China) according to standard protocols.

### Nucleotide Sequence Accession Number

Sequence data have been deposited under NCBI BioProject accession number PRJNA544155.

### Computational and Statistical Analyses of Bacterial Profiles

Clean data were extracted from raw data using USEARCH 8.0 (Edgar et al., 2011) with the following criteria: (i) sequences from each sample were extracted using each index with zero mismatch, (ii) sequences with an overlap of <50 bp were discarded, (iii) sequences in which the error rate of the overlap was >0.1 were discarded, and (iv) sequences <400 bp in length after the merge were discarded. Quality-filtered sequences were clustered into unique sequences and sorted in order of decreasing abundance to identify representative sequences using UPARSE (Edgar, 2013) according to the UPARSE operational taxonomic unit (OTU) analysis pipeline. Singletons were omitted in this step. OTUs were classified based on 97% similarity after removal of chimeric sequences using the UPARSE values (version 7.1^1^ ; Zhang Y. et al., 2015). The phylogenetic affiliation of each 16S rRNA gene sequence was analyzed using RDP Classifier^2^ (Wang et al., 2007) against the Silva (SSU132) 16S rRNA database with a confidence threshold of 70%.

Bacterial diversity was determined via sampling-based analysis of OTUs and displayed as a rarefaction curve. Bacterial richness and diversity across the samples were calculated using the following indexes: Chao1, ACE, and Shannon (Oksanen et al., 2015). To equalize the differences in sequencing depths among samples, the sequences were downsized to 5,000 per sample (20 permutations) (Zhang Q. et al., 2016). A non-parametric Mann–Whitney *U*-test was used to test for significant differences between two groups. Principal component analyses (PCAs) using weighted and unweighted UniFrac distance metrics were conducted, and the R package^3^ was used to visualize interactions among the bacterial communities from different samples (McMurdie and Holmes, 2013).

The specific characterization of fecal microbiota to distinguish taxonomic types was conducted using a linear discriminant analysis (LDA) effect size (LEfSe) method^4^ (Segata et al., 2011). Applying a normalized relative abundance matrix, LEfSe was used to: (i) identify key bacteria in fecal samples from the LT_A, LT_N, and HC groups at multiple levels in datasets, (ii) grade the key bacteria according to the results of a Mann–Whitney *U*-test, which determines features with significant differences in abundance between assigned taxa and uses LDA to assess the effect size of each feature (Ling et al., 2014), and (iii) visualize the results using taxonomic bar charts and cladograms. The *P*-values were adjusted as described by Benjamini and Hochberg (Jiang and Yu, 2017). Differences were considered significant when the false discovery rate was < 0.05.

Random forest models (Heitner et al., 2010) were introduced to identify key discriminatory OTUs between Group LT_A, LT_N, and Group HC. And to verify the key discriminatory OTUs which selected by random forest analysis, a 10-fold corss-validation analysis has been performed using rfcv function in R-package “randomForest” (R version 3.2.1). Firstly, random forest and Wilcox rank sum test were used to select differential species with both the value of Mean_decrease_in_accuracy above 0.001, and *p* < 0.05 by the Wilcox rank sum test (Zhang Q. et al., 2016); secondly, 10 times cross-validation analysis was performed to sift through the minimum OTU combination with the lowest error rate and the lowest number that can accurately separate the two groups; and receiver operating characteristics (ROC) analysis was then performed to measure of quality of the classification models by the R software package pROC (Robin et al., 2011). ROC curves were constructed, and the area under the curve (AUC) was used to designate the ROC effect.

Spearman’s correlation analyses were also used to assess potentially clinically relevant associations between the relative abundance of fecal bacterial genera and serum markers of liver dysfunction using Hmisc package in R.

## Results

### Clinical Characteristics of the Participants

After applying the strict inclusion and exclusion criteria described above, we enrolled 90 LT recipients (Group LT_A, *n* = 42; Group LT_N, *n* = 48) and 61 HC subjects (Group HC). There were no significant differences between the groups in terms of age, sex distribution, or body mass index. All liver recipients had previously been diagnosed as having hepatocellular carcinoma with cirrhosis, and all were HBV soluble antigen (HBsAg)-positive prior to LT. Serum levels of alanine aminotransferase, aspartate aminotransferase, and glutamyltranspeptidase were all significantly elevated in Group LT_A compared with Group HC and Group LT_N (all *P* < 0.001). Further details on the clinical characteristics of the subjects are provided in Table 1. Antibiotic treatments during the perioperative period (for at least 5 consecutive days) differed among patients because the use of prophylactic antimicrobial agents varied based on the perceived risks and willingness of the patients. The antimicrobial agents used included piperacillin-tazobactam, cefepime dihydrochloride, imipenem-cilastatin sodium, and micafungin sodium. The immunosuppressive therapies prescribed in the 6 months post-LT included simulect, mycophenolate mofetil, glucocorticoids, and FK506 tacrolimus; all drugs were used at doses adjusted according to patient situation based on several factors, including their patient status, platelet count, and white blood cell count. After 6 months post-LT, FK506 tacrolimus (at a serum level of 5–6 ng/ml) with or without mycophenolate mofetil (1 g/day) was used. General liver protective drugs combined with appropriately increasing dosage of anti-immunosuppressive agents were treated in those recipients of Group LT_A. And prophylaxis with high-dose hepatitis B immunoglobulin (HBIG) and Entecavir [nucleos(t)ide analogs] was used after the transplantation to suppress viral replication. As follows: for each recipient, entecavir capsule (oral medication): one capsule daily for life; and HBIG: 2000 intl units/day through intravenous drip within postoperative week 1, 2000 intl units/week through intravenous drip within postoperative week 2–5, then 800 intl units twice a week though intramuscular injection if patients fail to reach anti-HBs levels of 100 intl units/L. The whole blood concentration of HBIG after liver transplantation is needed to monitor for life. If patients fail to reach anti-HBs levels of 500 intl units/L, which tested in the 2th day after using HBIG, within the first month post-liver transplantation, dosage adjustments would be required. We enrolled recipients who were in the post-LT period of >24 months and <48 months because patients were in a stable condition during this period and were not exposed to antibiotics in the 12 weeks prior to participation in the study. In addition, all LT recipients only took FK506 tacrolimus as an immunosuppressive therapy during this period.

**TABLE 1:** Clinical information in liver recipients and healthy controls.

**Clinical and pathological Indexes**		**Group LT_A**		**Group LT_N**		**Healthy controls**			***P*-value**	
			
		***N* = 42**	**%**	***N* = 48**	**%**	***N* = 61**	**%**	**HC vs. LT_A**	**HC vs. LT_N**	**LT_A vs. LT_N**
Gender	Female	10	23.81	10	20.83	15	24.59	0.999	0.819	0.802
	Male	32	76.19	38	79.17	46	75.41			
Age (year)		46.62 ± 1.51		46.90 ± 1.36		48.21 ± 0.84		0.324	0.391	0.892
BMI (kg/m^2^)		23.72 ± 0.32		23.50 ± 0.41		23.72 ± 0.32		0.174	0.432	0.677
Time of post-LT (month)		28.05 ± 2.21		32.79 ± 2.02		–				0.117
ALT (5–40 U/L)		105.12 ± 21.96		22.15 ± 1.03		20.56 ± 1.05		1.047E-05	0.289	0.0001
AST (8–40 U/L)		68.38 ± 8.76		22.17 ± 0.69		22.16 ± 0.60		6.39E-09	0.998	2.165E-07
GGT (11–50 U/L)		121.74 ± 19.37		43.98 ± 4.87		22.59 ± 1.79		1.733E-08	1.77E-05	8.297E-05
Total bilirubin (0–21 μmol/L)		119.67 ± 23.77		15.33 ± 0.86		13.79 ± 0.70		4.926E-07	0.162	9.826E-06

**The continuous variables were presented as mean ± standard error of mean (SEM); the continuous variables were compared with the independent t-test between both groups. Fisher’s exact test was used to compare categorical variables. – without LT. Group LT_A, liver transplantation recipients with abnormal liver function; Group LT_N, liver transplantation recipients with normal liver function; HC, healthy controls. BMI, body mass index; ALT, alanine aminotransferase; AST, aspartate aminotransferase; GGT, glutamyl transpeptidase.**

### Recipients in Group LT_A Showed Decreased Fecal Microbial Diversity

**FIGURE 1:**
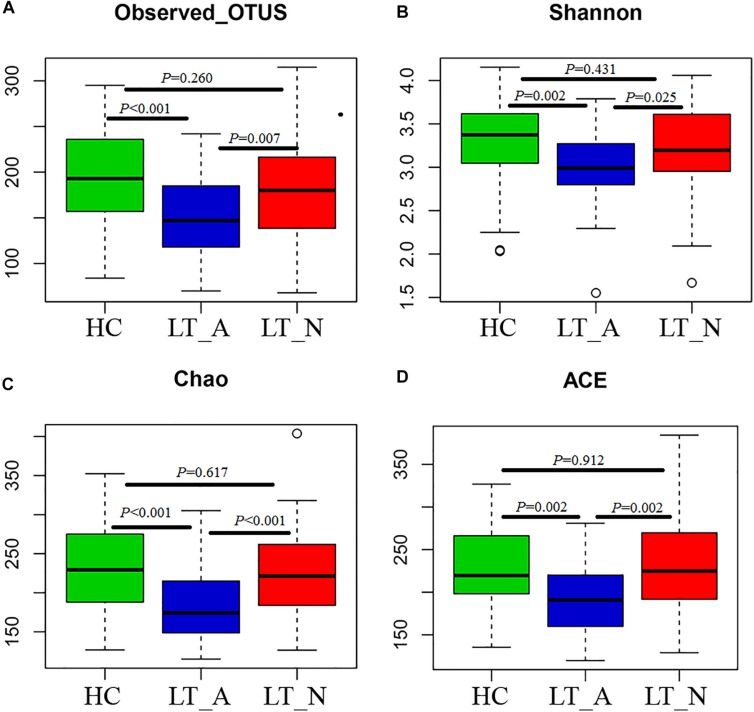
Phylogenetic diversity of fecal microbiomes between liver transplant recipients and healthy subjects. **(A–D)** Box plots depicting microbiome diversity differences according to the number of OTUs detected, the Shannon index, the Chao 1 index, and the ACE index, respectively. “+” represents the median value, while the upper and lower ranges of the box represent the 75 and 25% quartiles, respectively. LT_A, fecal microbiomes of the liver recipients with abnormal liver function; LT_N, fecal microbiomes of the liver recipients with normal liver function; HC, fecal microbiomes of the healthy control group; OTU, operational taxonomic unit.

We generated 4,723,696 filtered sequences from the fecal samples of 90 LT recipients and 61 HCs. The qualified reads were clustered into 588 qualified species-level OTUs using 97% as the similarity cutoff. Overall, 88.3 and 60.4% of all reads could be assigned to the family and genus levels, respectively (Supplementary Dataset S1A). Species-level OTUs and species richness and diversity estimates were obtained for each microbiome (Supplementary Dataset S1B). The species richness of individual samples and of the total gut bacterial communities was estimated by rarefaction analysis. The resulting rarefaction curves indicated that the microbial richness of the sampled guts was near saturation at the applied sequencing depth (Supplementary Figures S1A,B), which was sufficient to identify most of the bacterial community members of each individual microbiome. Fecal microbiome estimated richness in ascending order was Group LT_A, Group LT_N, and Group HC (Supplementary Figure S1C). The abundance of OTUs in Group LT_A was significantly lower than that in Group LT_N and Group HC (*P* = 0.007 and <0.001, respectively, Figure 1A). Additionally, only 78.2% (460 bacterial OTUs) of the microbiome was shared by the three groups, with 35, 2, and 18 bacterial OTUs unique to Group HC, Group LT_A, and Group LT_N, respectively (Supplementary Figure S1D).

Subjects in Group LT_A exhibited an obviously different fecal microbiome composition compared with those in Groups LT_N and HC. In particular, the microbial diversity was significantly decreased in Group LT_A compared with the other two groups (*P* = 0.025 and 0.002, respectively), as estimated by the Shannon index (Figure 1B). This finding was validated by the other applied diversity parameters, namely the Chao1 and ACE indexes (Chao1 index: *P* < 0.000 vs. Group LT_N and *P* < 0.000 vs. Group HC, Figure 1C; ACE index: *P* = 0.002 vs. Group LT_N and *P* = 0.002 vs. Group HC, Figure 1D).

**FIGURE 2:**
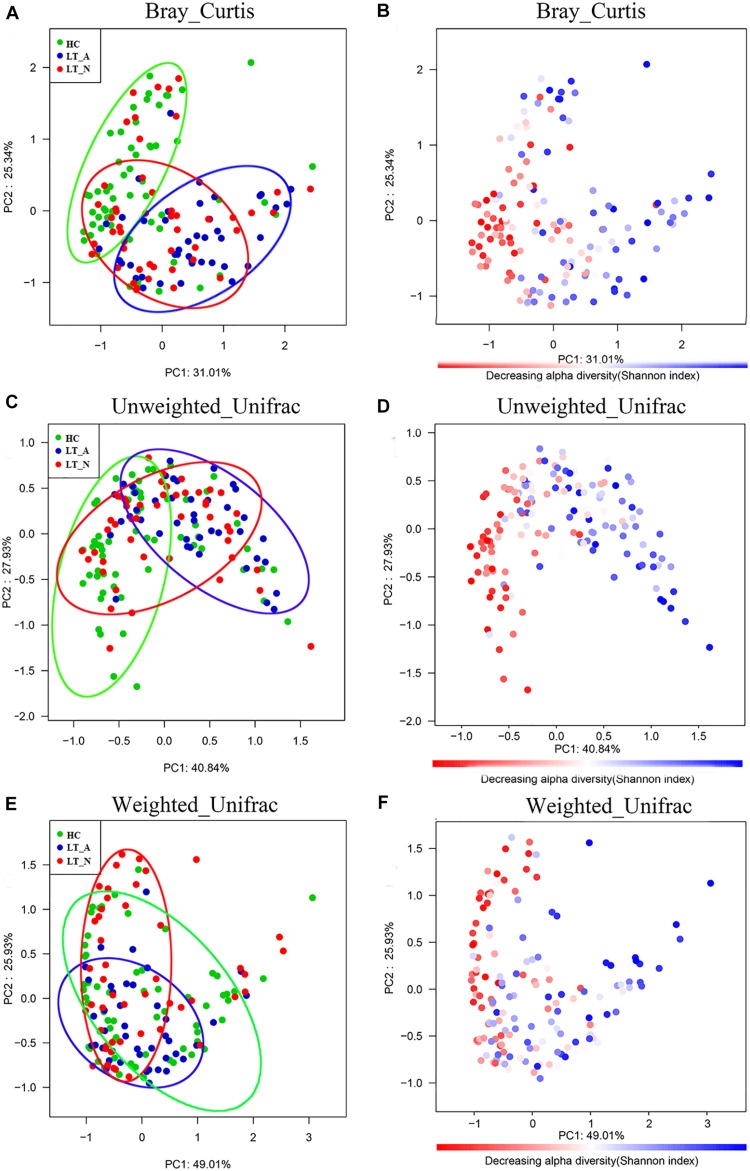
Bacterial diversity clustering determined by combining the results of Bray-Curtis analysis and unweighted and weighted UniFrac PCoA of fecal microbiota. The beta diversity is different between LT_A and HC, and LT_A and LT_N (*P* < 0.05 using PERMANOVA analyses with R-vegan function Adonis). The variance explained by the principal components is indicated in parentheses on the axis, and the ellipses highlight the clustering of the fecal microbiomes according to groups (green, Group HC; blue, Group LT_A; red, Group LT_N). **(A,B)** Indicate the results of Bray-Curtis analysis, **(C,D)** are the results based on unweighted UniFrac (qualitative) analysis, and **(E,F)** show the results of weighted UniFrac (qualitative) analysis. Each point represents a sample. **(A,C,E)** The samples from Group LT_A, Group LT_N, and Group HC are represented in blue, red, and green, respectively. **(B,D,F)** Microbial diversity maps. A highly diverse core (colored in red) indicates the bacterial diversity of Group HC is highest among the three groups (the color scale of red to blue reflects the decreasing alpha diversity of the fecal microbiome). LT_A, fecal microbiomes of the liver recipients with abnormal liver function; LT_N, fecal microbiomes of the liver recipients with normal liver function; HC, fecal microbiomes of the healthy control group; PCoA, principal coordinates analysis.

The Bray-Curtis (OTU number dissimilarity, Figures 2A,B), unweighted-UniFrac (qualitative, Figures 2C,D), and weighted-UniFrac (quantitative, Figures 2E,F) PCA plots, which measure the phylogenetic similarities between microbial communities, showed that the fecal microbiota of subjects in Group LT_A differed from that of the HCs, while the fecal microbiota of subjects in Group LT_N overlapped with that of subjects belonging to Groups LT_A and HC. The lower fecal microbiome diversity in Group LT compared with Group HC is also depicted in the PCA plots (Figures 2B,D,F).

### Bacterial Taxonomic Differences Between LT Recipient Groups and HCs

Many of the taxa were differentially abundant in LT_A, LT_N, and HC. Analysis at the class level (Supplementary Figure S2A) showed that the relative abundance of Negativicutes, Gammaprotobacteria and Bacilli were significantly higher in both the LT_N and LT_A groups compared with the HC group; additionally, Fusobacteriia were significantly increased, and Firmicutes_unclassified and Lentisphaerae were significantly decreased in LT_A when compared with HC. We also performed comparisons at the phylum level (Supplementary Figure S2B) between the patient groups and healthy controls, and found that Firmicutes were significantly decreased in both the LT_N and LT_A groups compared with the HC group. Interestingly, when compared with healthy controls, LT_A showed higher relative abundance of Proteobacteria and Fusobacteria, while LT_N showed higher relative abundance of Bacteroidetes.

**FIGURE 3:**
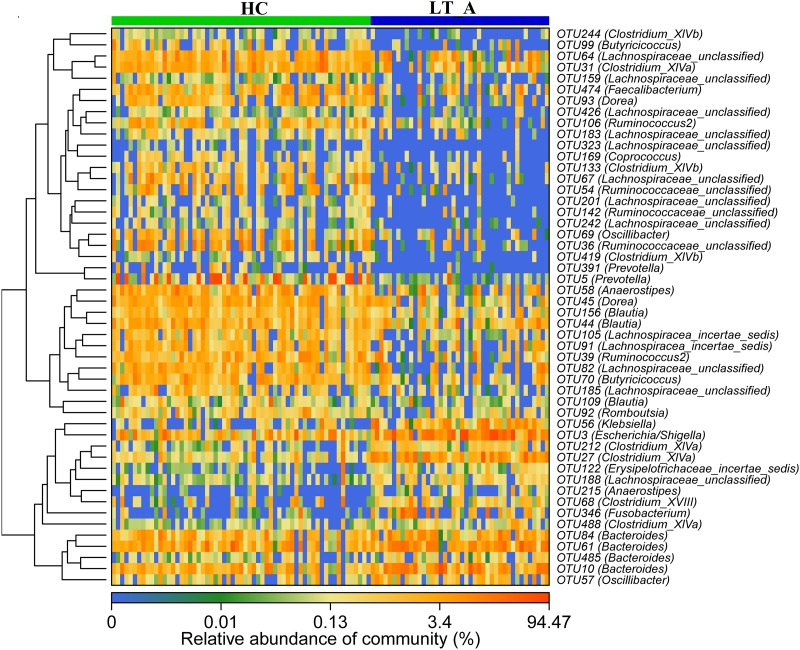
Heat maps showing the relative abundance of the discriminatory OTUs that drive the differences between groups LT_A and HC. For each sample, the columns show the relative abundance data for the discriminatory OTUs listed to the right of the figure. Abundance values for each of the genera were clustered using unsupervised hierarchical clustering [the relative abundance of each genus is indicated by a gradient of color from blue (low abundance) to red (high abundance)]. The corresponding genus of each key OTU is noted to the right of the figure. The heat map on the left shows Spearman hierarchical clustering of relative abundance values for each of the 50 most discriminatory 97%-identity OTUs in a random forest-based model of the fecal microbiota of the LT_A and HC groups. LT_A, fecal microbiomes of the liver recipients with abnormal liver function; HC, fecal microbiomes of the healthy control group; OTU, operational taxonomic unit.

The results of heatmap analysis (a hierarchical clustering analysis) of the fecal microbiomes using a random forests model revealed a discriminatory intestinal microbiome between LT recipient groups and healthy subjects. Comparison of the Group LT_A and Group HC fecal microbiomes revealed 50–97%-identity OTUs assigned to 20 different families/genera that were significantly differently distributed between the two groups (Figure 3). Of these 50 OTUs, 35 OTUs corresponding to the families/genera *Butyricicoccus* (2 OTUs), *Prevotella* (2 OTUs), *Clostridium*_XIVb (3 OTUs), *Clostridium_*XIVa (1 OTU), *Lachnospiraceae* (12 OTUs), *Faecalibacterium* (1 OTU), *Dorea* (2 OTUs), *Ruminococcaceae* (5 OTUs), *Romboutsia* (1 OTU), *Anaerostipes* (1 OTU), *Coprococcus* (1 OTU), *Blautia* (3 OTUs), and *Oscillibacter* (1 OTU) were more abundant in Group HC than in Group LT_A. The remaining OTUs, including those corresponding to *Klebsiella* (1 OTU), *Escherichia*/*Shigella* (1 OTU), *Clostridium*_XIVa (3 OTUs), *Bacteroides* (4 OTUs), *Fusobacterium* (1 OTU), *Lachnospiraceae* (1 OTU), *Anaerostipes* (1 OTU), *Erysipelotrichaceae*_incertae_sedis (1 OTU), and *Clostridium*_XVIII (1 OTU) were more abundant in Group LT_A than in Group HC.

**FIGURE 4:**
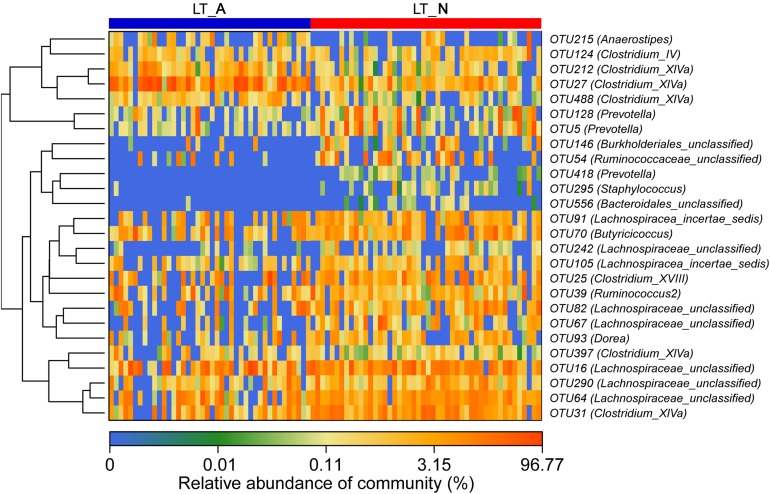
Heat maps showing the relative abundance of the discriminatory OTUs that drive the differences between groups LT_A and LT_N. For each sample, the columns show the relative abundance data for the discriminatory OTUs listed to the right of the figure. The abundance values for each of the genera were clustered using unsupervised hierarchical clustering [the relative abundance of each genus is indicated by a gradient of color from blue (low abundance) to red (high abundance)]. The corresponding genus of each key OTU is noted to the right of the figure. The heat map on the left shows Spearman hierarchical clustering of relative abundance values for each of the 26 most discriminatory 97%-identity OTUs in a random forest-based model of the fecal microbiota of groups LT_A and LT_N. LT_A, fecal microbiomes of the liver recipients with abnormal liver function; LT_N, fecal microbiomes of the liver recipients with normal liver function; OTU, operational taxonomic unit.

Comparison of the Group LT_A and Group LT_N fecal microbiomes revealed 26 most-discriminatory-OTUs. Of these, five OTUs, corresponding to *Anaerostipes* (1 OTU), *Clostridium*_IV (1 OTU), and *Clostridium*_XIVa (3 OTUs), were more abundant and 21 OTUs, including *Prevotella* (3 OTUs), *Staphylococcus* (1 OTU), *Burkholderiales* (1 OTU), *Clostridium*_XIVa (2 OTUs), *Ruminococcaceae* (2 OTUs), *Lachnospiracea* (8 OTUs), *Bacteroidales* (1 OTU), *Clostridium*_XVIII (1 OTU), *Butyricicoccus* (1 OTU), and *Dorea* (1 OTU), were less abundant in Group LT_A than in Group LT_N (Figure 4). Additionally, heatmap analysis of the fecal microbiomes delineated 29 distinguishing OTUs, which were assigned to 18 different genera, between groups LT_N and HC. Of these most-discriminatory-OTUs, 14 OTUs corresponding to *Lachnospiraceae* (7 OTUs), *Dorea* (1 OTU), *Clostridium*_XIVb (1 OTU), *Ruminococcus* 2 (1 OTU), Firmicutes (1 OTU), *Victivallis* (1 OTU), and *Bacteroides* (2 OTUs) were decreased, while 15 OTUs, including *Megamonas* (1 OTU), *Prevotella* (1 OTU), *Lactobacillus* (1 OTU), *Enterococcus* (1 OTU), *Klebsiella* (1 OTU), *Veillonella* (2 OTUs), *Streptococccus* (1 OTU), *Clostridium*_XIVa (1 OTU), *Bacteroides* (2 OTUs), *Erysipelotrichaceae*_incertae_sedis (1 OTU), *Ruminococcaceae* (1 OTU), and *Lachnospiraceae* (2 OTUs), were increased in the fecal microbiome of Group LT_N compared with that of Group HC (Supplementary Figure S3).

**FIGURE 5:**
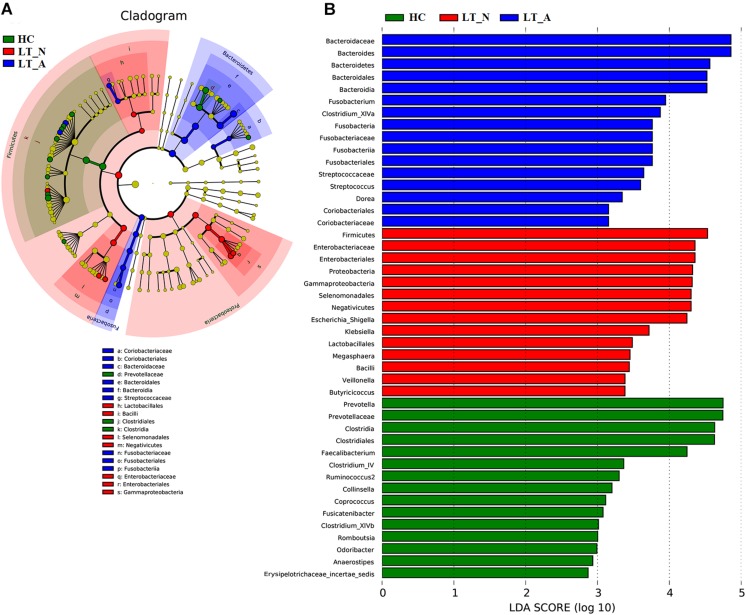
LEfSe and LDA based on divergent OTUs between the fecal microbiomes of the LT_A, LT_N, and HC groups, which identified the most differentially-abundant taxa between the three groups. **(A)** Taxonomic cladogram generated using the LEfSe method. LT_A-enriched taxa, blue; LT_N-enriched taxa, red; taxa enriched in healthy controls, green. **(B)** LDA scores indicating significant differences in microbiota between the three groups (LT_A, LT_N, and HC). HC-enriched taxa, green; LT_A-enriched taxa, blue; LT_N-enriched taxa, red. LT_A, fecal microbiomes of the liver recipients with abnormal liver function; LT_N, fecal microbiomes of the liver recipients with normal liver function; HC, fecal microbiomes of the healthy control group; LEfSE, linear discriminant analysis effect size; LDA, linear discriminant analysis; OTU, operational taxonomic unit.

We also used LEfSe to compare the estimated phylotypes of the recipient groups with the Group HC microbiota (Figures 5A,B), with the results confirming that dysbiosis was indeed present at various phylogenetic levels. At the family level, the Group LT_A fecal microbiome was characterized by a preponderance of *Bacteroidaceae*, *Fusobacteriaceae*, *Streptococcaceae*, *Coriobacteriaceae*, and *Lachnospiraceae* (all LDA scores (log_10_) >3 and *P* < 0.05; Supplementary Dataset S2), while the Group LT_N fecal microbiome was characterized by a higher prevalence of *Enterobacteriaceae*, *Veillonellaceae*, and *Ruminococcaceae* (all LDA scores (log_10_) > 3 and *P* < 0.05; Supplementary Dataset S2). In comparison, the HC fecal microbiomes were dominated by *Prevotellaceae*, *Porphyromonadaceae*, *Lachnospiraceae*, *Coriobacteriaceae*, *Peptostreptococcaceae*, *Ruminococcaceae*, and *Erysipelotrichaceae* (all LDA scores (log_10_) > 3 and *P* < 0.05; Supplementary Dataset S2). At the genus level, opportunistic pathogens (including *Bacteroides*, *Fusobacterium*, and *Streptococcus*) and butyrate-producing bacteria (including *Clostridium*_XIVa and *Dorea*) were enriched in the Group LT_A fecal microbiome (all LDA scores (log_10_) > 3 and *P* < 0.05; Supplementary Dataset S2), while opportunistic pathogens (including *Escherichia_Shigella*, *Klebsiella*, and *Veillonella*), *Megasphaera* (lactate-utilizing bacteria), and *Butyricicoccus* (butyrate-producing bacteria) were enriched in the Group LT_N fecal microbiome (all LDA scores (log_10_) > 3 and *P* < 0.05; Supplementary Dataset S2). In comparison, *Prevotella*, *Collinsella*, *Romboutsia*, *Odoribacter*, and butyrate-producing bacteria (including *Faecalibacterium*, *Clostridium*_IV, *Ruminococcus* 2, *Coprococcus*, *Fusicatenibacter*, and *Clostridium*_XIVb) were enriched in the HC microbiome (all LDA scores (log_10_) > 3 and *P* < 0.05; Supplementary Table S2).

**FIGURE 6:**
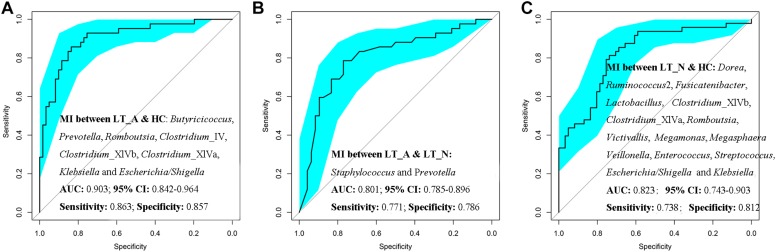
Prediction of the key genera (microbiotal index, MI) in the fecal microbiomes of LT_A, LT_N, and HC. **(A)** ROC curves for *Prevotella*, *Romboutsia*, *Klebsiella*, *Escherichia_Shigella*, *Butyricicoccus*, *Clostridium*_IV, *Clostridium*_XIVb, and *Clostridium*_XIVa could be used to distinguish samples belonging to Group LT_A from those of Group HC, with ROC plot AUC values of 0.903 and sensitivity and specificity values of 0.812 and 0.690, respectively. **(B)** ROC plots for *Staphylococcus* and *Prevotella* could distinguish fecal microbiomes of Group LT_A recipients from those of Group LT_N, with ROC-AUC values of 0.801 and sensitivity and specificity values of 0.771 and 0.786, respectively. **(C)** ROC plots for *Victivallis*, *Romboutsia*, *Megasphaera*, *Megamonas*, *Veillonella*, *Lactobacillus*, *Klebsiella*, *Escherichia/Shigella*, *Enterococcus*, *Streptococcus*, *Ruminococcus* 2, *Fusicatenibacter*, *Dorea*, *Clostridium_*XIVb, and *Clostridium* XIVa could distinguish fecal microbiomes from Group LT_N recipients from those of Group HC, with ROC-AUC values of 0.823 and sensitivity and specificity values of 0.738 and 0.812, respectively. LT_A, fecal microbiomes of the liver recipients with abnormal liver function; LT_N, fecal microbiomes of the liver recipients with normal liver function; HC, fecal microbiomes of the healthy control group; ROC, receiver operating characteristic; AUC, the area under the parasitemia curve.

Additionally, *Prevotella*, *Romboutsia*, opportunistic pathogens (including *Klebsiella* and *Escherichia_Shigella*), and butyrate-producing bacteria (including *Butyricicoccus*, *Clostridium*_IV, *Clostridium*_XIVb, and *Clostridium*_XIVa) could be used to distinguish Group LT_A samples from those belonging to Group HC, with ROC-plot AUC values of 0.903 and sensitivity and specificity values of 0.863 and 0.857, respectively **(**Figure 6A; ROC-AUC values shown in Supplementary Dataset S2A), while *Staphylococcus* and *Prevotella* could be used to distinguish the fecal microbiomes of Group LT_A from those of Group LT_N, with ROC-AUC values of 0.801 and sensitivity and specificity values of 0.771 and 0.786, respectively (Figure 6B; ROC-AUC values shown in Supplementary Dataset S2B). Further, *Victivallis*, *Romboutsia*, *Megasphaera*, *Megamonas*, *Veillonella*, *Lactobacillus*, opportunistic pathogens (including *Klebsiella*, *Escherichia/Shigella*, *Enterococcus*, and *Streptococcus*), and butyrate-producing bacteria (including *Ruminococcus* 2, *Fusicatenibacter*, *Dorea*, *Clostridium_*XIVb, and XIVa) could be used to distinguish fecal microbiomes of Group LT_N recipients from those of Group HC, with ROC-AUC values of 0.823 and sensitivity and specificity values of 0.738 and 0.812, respectively (Figure 6C; ROC_AUC values shown in Supplementary Dataset S2C).

Next to better understand the profile of LT-associated fecal microbiome, we computed covariations between the relative abundance of the discriminatory fecal bacterial genera, and between these genera and clinical indices for liver function including ALT, AST, TB, and GGT (Supplementary Figure S4), and we noted that bacteria in most LT_A-associated bacteria including *Bacteroides*, *Fusobacterium*, *Clostridium*_XIVa, *Streptococcus*, and opportunistic pathogens (including *Klebsiella* and *Escherichia_Shigella*) especially *Bacteroides* and *Klebsiella* (Spearman’s correlation >0.3, *p* < 0.05 for the correlations of both genera with all serum markers; Supplementary Figure S4), positively correlated with serum markers of liver dysfunction; while HC-associated bacteria including *Butyricicoccus*, *Clostridium*_IV, *Clostridium*_XIVb, *Prevotella*, *Collinsella*, *Romboutsia*, *Faecalibacterium*, *Ruminococcus* 2, *Coprococcus*, *Fusicatenibacter*, especially *Butyricicoccus* and *Prevotella* (Spearman’s correlation <-0.3, *p* < 0.05 for the correlations of both genera with all serum markers; Supplementary Figure S4), negatively correlated with serum markers of liver dysfunction.

## Discussion

The host immune system controls the composition, diversity, and location of the microbiota (Thaiss et al., 2016). Alterations in the intestinal microbiome are associated with changes in immunity and metabolism, which play critical roles in the pathogenesis of human liver diseases. Therefore, the restoration of the post-LT microbiota is a dynamic process between the host immune system and bacterial colonization. Mounting evidence has shown that disruption of this complex and delicate homeostasis may worsen liver pathogenetic conditions. The restoration of the microbiota in the early-stages of post-LT recovery is important for long-term complication management (Doycheva et al., 2016). Our previous large, clinical cohort study explored changes to the six predominant gut bacterial genera and to the immune indices of patients who underwent liver transplantation (Wu et al., 2012). That work was the first to report the dysbiosis of gut microbiota in subjects undergoing liver transplantation. Here, we used high-throughput sequencing platforms to identify key bacterial families and genera in the fecal microbiomes of liver recipient cohorts with normal/abnormal liver function, which may help provide targets for microbiota restoration following surgery and immunosuppressant treatments. As pre-transplant intestinal microbiota dysbiosis in LT recipients has a more powerful influence on the post-transplant microbiota than the LT itself (Xie et al., 2011), the present study only included recipients who were diagnosed with HBV-associated hepatocellular carcinoma.

An abundance of opportunistic pathogens in the intestinal microbiota may be detrimental to long-term health after LT. Among the 20 most-discriminatory bacteria enriched in the liver recipients, several are opportunistic pathogens, including *Fusobacterium* (Aliyu et al., 2004), *Streptococcus*, *Klebsiella*, and *Escherichia/Shigella*. Although *Fusobacterium* species are members of the normal gut microbiota of humans, certain species (adherent, invasive, and/or pro-inflammatory) are recognized as opportunistic pathogens and are enriched in the gut microbiomes of patients with liver cirrhosis (Qin et al., 2014) and liver cancer (Ren et al., 2018). *Streptococcus* species, which form part of the commensal human intestinal microbiota, are also enriched in the intestinal microbiomes of patients with liver cirrhosis (Qin et al., 2014). In the present study, both genera were enriched in the fecal microbiomes of recipients with abnormal liver function. *Enterobacteriaceae* enrichment in the disturbed microbiota is associated with an increase of endotoxin production, which leading to endotoxemia, increased intestinal permeability, and liver injury (Arai et al., 1998). There is a positive correlation between overgrowth of *Enterobacteriaceae* and the development of liver dysfunction, as Gram-negative bacterial cell components such as lipopolysaccharide induce over-expression of pro-inflammatory cytokines, chemokines, and some reactive oxygen/nitrogen species by luminal epithelial cells and Kupffer cells (Racanelli and Rehermann, 2006; Szabo and Bala, 2010). In present study, we found relative abundance of opportunistic pathogens had a positive correlation with serum markers of liver dysfunction. The finding that the relative abundance of *Klebsiella* and *Escherichia/Shigella* was much higher in the recipients with normal liver function than in those with abnormal liver function and in the HCs indicates the need for personalized management of individual patients post-transplant.

Our compositional analysis of the fecal microbiota of both LT groups and HCs suggests that the major difference between the three groups is not in the composition of opportunistic bacteria, but rather, in butyrate-producing bacteria. *Lachnospiraceae*, *Odoribacteraceae*, and some clusters of *Clostridiaceae* are known butyrate producers. We found that the recipient groups showed less diversity in butyrate-producing bacteria compared with HCs. *Clostridium* cluster IV (*Faecalibacterium* and *Ruminococcus*) (Schwiertz et al., 2002b; Fang et al., 2017), *Clostridium* cluster XI (*Anaerostipes*) (Schwiertz et al., 2002a; Kant et al., 2015), *Clostridium* cluster XIVb, *Clostridium* cluster XIVa (*Coprococcus* and *Lachnospiraceae*), and *Odoribacter* (belonging to *Odoribacteraceae*) (Gomez-Arango et al., 2016; Callejo et al., 2018) were enriched in control subjects, while *Dorea* (belong to *Clostridium* cluster XIVa) and *Butyricicoccus* (belonging to *Clostridium* cluster IV) were enriched in Groups LT_A and LT_N, respectively. Evidence has shown that butyrate is essential for the maintenance of colonic mucosal health, with roles such as inducing the development of regulatory T cells (Furusawa et al., 2013) and regulating the Treg/Th17 balance, which helps to restore intestinal homeostasis (Zhang M. et al., 2016). *Clostridium* clusters XIVa and IV are reported to stimulate over-expression of interleukin 10 (IL-10) and cytotoxic T lymphocyte-associated antigen 4 (CTLA4) by colon Treg cells (Atarashi et al., 2011). Over-expression of IL-10 and CTLA4 plays an important role in the development of liver injury (Knolle and Gerken, 2000), graft rejection, and in the long-term clinical outcome of organ transplantation patients (Alegre et al., 2001; Guo et al., 2012). Additionally, a decreased abundance of butyrate-producing bacteria (*Ruminococcaceae* and *Clostridiales* family XI Incertae Sedis) was also observed in patients with inflammatory bowel disease, cirrhosis, and early hepatocellular carcinoma compared with controls (Chen et al., 2011; Joossens et al., 2011; Ren et al., 2018). Butyrate mediates suppression of inflammation and carcinogenesis by interacting with metabolite-sensing G protein-coupled receptors in gut epithelial and immune cells (Sivaprakasam et al., 2016). In present study, the relative abundance of fecal butyrate-producing bacteria had a negative correlation with serum markers of liver dysfunction. Therefore, dysbiosis of intestinal butyrate-producing bacterial populations might result in the progression of chronic liver injury in the liver recipient cohorts.

Although the shifts in the intestinal microbiome of LT recipients under the pressure of long-term immunosuppressant use are uncertain, the known relationship between the host immune system and the intestinal microbiota suggests that immunosuppressors likely affect the repopulation of the intestinal microbiome in LT recipients. Additionally, antibiotic treatments to prevent or eradicate a pathogen are likely to have both short- and long-term impacts on the commensal microbiota (Jernberg et al., 2010; Sommer and Dantas, 2011). These effects can change the relative proportions of different species in the microbiota, with the introduction of a new species or the eradication of an existing species. Finally, the intestinal microbiome and the liver are closely connected (Henao-Mejia et al., 2013); thus, abnormal liver function in LT recipients also contributes to the perturbation of microbiota-host mutualism. The occurrence and development of liver disease are always accompanied by intestinal microbial variation, while structural shifts in the intestinal microbiota always contribute to liver injury or its recovery following hepatic surgery (Ren et al., 2011). Our previous study showed that fecal bacterial populations could serve as non-invasive independent biomarkers for early detection of hepatocellular carcinoma (Ren et al., 2018). Previous animal experiments revealed that alterations in the intestinal microbiota predated hepatic rejection injury in rats following LT (Ren et al., 2014), suggesting that intestinal microbial variation might predict early acute rejection after LT and could become a therapeutic target to improve rejection rates after orthotopic LT. Chronic rejection following LT was always accompanied by abnormal liver function. Here, the fecal microbiota heat map and LEfSe analysis revealed that patients in group LT_A had serious fecal microbiome dysbiosis, with lower butyrate-producing bacterial diversity and a higher prevalence of opportunistic pathogens compared with HCs and liver recipients with normal liver function. Interestingly, enrichment of *Klebsiella* and *Escherichia/Shigella* was observed in liver recipients from both groups compared with HCs.

The gut microbiota is increasingly being recognized as an attractive target for therapeutic intervention, and stimulation of butyric acid production in the intestine of LT recipients could be achieved via the repopulation of butyrate-producers through microbiota-targeted therapies. This novel strategy could reduce chronic liver allograft dysfunction and other complications (Loguercio et al., 2005), as well as improve the quality of life for LT recipients. Understanding the ecological and evolutionary processes that determine the diversity and composition of the repopulated intestinal microbiome in LT recipients with abnormal/normal liver function is a critical first step for achieving these goals.

It should be noted that our findings on the complex discriminatory fecal microbiomes of both LT groups and HCs is only a snapshot, and that we only analyzed the bacterial composition of the fecal microbiota. As such, the current study has several limitations. First, this study didn’t include the microbiota profiling prior to liver transplantation. Although we could enroll HBV-associated hepatocellular carcinoma patients with similar pathophysiology and disease severity, patients could not be asked to receive the same medical interventions and treatment within perioperative period, because the use of prophylactic antimicrobial and immunosuppressant agents varied by the targeted disease and the recipient’s perceived risks and willingness. Second, the present study was not designed to compare microbiota alterations before and after orthotopic LT surgeries. Future long-term follow-up microbiome studies including pre- and post-orthotopic LT recipients should include multiple cohorts and a later post-operative sampling point to characterize the architecture of a healthy LT recipient microbiome.

In summary, these results demonstrated that the variations in fecal microbiota profiles of LT patients, and identified fecal butyrate-producing bacteria enriched in healthy control samples were negatively correlated with serum markers of liver dysfunction. Lastly, microbiota-directed therapeutic strategies should be considered after orthotopic LT surgeries.

## Ethics Statement

This study was approved by the Institutional Review Board of the First Affiliated Hospital, School of Medicine, Zhejiang University (IRB No. 2014-336). All experiments were performed in accordance with the Helsinki Declaration and the Rules of Good Clinical Practice, and no organs from executed prisoners were used in these recipients.

## Author Contributions

L-JL and S-SZ conceived and designed the experiments. H-FL, Z-GR, HZ, S-YX, J-WJ, LZ, QL, and B-HW performed the experiments. H-FL, Z-GR, AL, G-YC, and X-HC analyzed the data. LZ, S-SZ, and L-JL contributed reagents, materials, and analysis tools. Z-GR prepared the figures and the tables. H-FL wrote the manuscript.

## Conflict of Interest Statement

The authors declare that the research was conducted in the absence of any commercial or financial relationships that could be construed as a potential conflict of interest.

## Footnotes

1 http://drive5.com/uparse/

2 http://rdp.cme.msu.edu/

3 http://www.R-project.org/

4 http://huttenhower.sph.harvard.edu/lefse/

## Abbreviations

AUC: the area under the parasitemia curve
HC: healthy controls
HE: hepatic encephalopathy
LDA: linear discriminant analysis
LT: liver transplant
MELD: model for end-stage liver disease
OTUs: operational taxonomic units
PICRUSt: Phylogenetic Investigation of Communities by Reconstruction of Unobserved States software
ROC: receiver operating characteristic curve
rRNA: ribosomal RNA
SCFA: short chain fatty acids.
