# Supplementary material for: Fecal Microbiome Data Distinguish Liver Recipients With Normal and Abnormal Liver Function From Healthy Controls
Source: Front Microbiol. 2019 Jul 3;10:1518. doi: 10.3389/fmicb.2019.01518 (PMC6619441; doi:10.3389/fmicb.2019.01518)
Supplement: Supplementary file 1 [file Data_Sheet_1.docx]

**Title:** **Fecal** **Microbiome Data Distinguish Liver Recipients with Normal and Abnormal Liver Function from Healthy Controls.**

Hai-Feng Lu^1ǂ^, Zhi-Gang Ren^2,3,ǂ^, Ang Li^1,2^, Hua Zhang^1^, Shao-Yan Xu^3^, Jian-Wen Jiang^3,4^, Lin Zhou^3^, Qi Ling^3^, Bao-Hong Wang^1^, Guang-Ying Cui^2^, Xin-Hua Chen^3^, Shu-Sen Zheng^3*^, Lan-Juan Li^1*^

^1^State Key Laboratory for Diagnosis and Treatment of Infectious Disease, Collaborative Innovation Center for Diagnosis and Treatment of Infectious Diseases, the First Affiliated Hospital, School of Medicine, Zhejiang University, Hangzhou 310003, P.R. China.

^2^Department of Infectious Diseases, Precision Medicine Center, the First Affiliated Hospital of Zhengzhou University, Zhengzhou 450052, P.R. China.

^3^Department of Hepatobiliary and Pancreatic Surgery, the First Affiliated Hospital, School of Medicine, Zhejiang University; Key Laboratory of Combined Multi-organ Transplantation, Ministry of Public Health, Hangzhou 310003, P.R. China.

^4^Health Management Center, the First Affiliated Hospital, School of Medicine, Zhejiang University, Hangzhou 310003, China.

ǂThese authors contributed equally to this work.

***Corresponding authors**: Professor Lanjuan Li, State Key Laboratory for Diagnosis and Treatment of Infectious Disease, the First Affiliated Hospital, School of Medicine, Zhejiang University, Hangzhou 310003, China; ljli@zju.edu.cn; and Professor Shusen Zheng, Department of Hepatobiliary and Pancreatic Surgery, the First Affiliated Hospital, School of Medicine, Zhejiang University, Hangzhou 310003, China; shusenzheng@ zju. edu. cn,

**Supplementary information**

**Supplementary Table S1** Questionnaire: Data for subjects participated in the investigation of the fecal microbiome.

**Data for subjects participated in the investigation of the fecal microbiome**

**Name： sex：F/M Date of birth (**month-year**)：**

**Telephone number(s)： Sample NO:**

| Data of follow-up(Y-M-D) | | |  | | | | |
| --- | --- | --- | --- | --- | --- | --- | --- |
| Way of follow- up | | | 1. outpatient 2 inpatient | | | | |
| A survey   of disease symptom | 1.Liver, lung or previous related surgery  2.Basic diseases such as diabetes, coronary disease,hypertension  3. Ascites  4.Gastrointestinal bleeding  5.Liver cirrhosis  6.Co-infection with HCV, HIV or other secondary bacterial infection  7.Symptoms of respiratory: shortness of breath, sputum production,wheezing or chest pain or discomfort.  8.Oothers | | Pre- LT surgery | | Post-LT surgery | | |
|  |  |  |  | |  | | |
| Ultrasonography | | | Examation day： | | | | |
|  |  |  |  | | | | |
| Biochemical blood routine inspection | | | Examation day： | | | | |
|  |  |  | HBsAg: | | | | |
|  |  |  | Alanine aminotransferase: | | | | |
|  |  |  | Aspartate aminotransferase: | | | | |
|  |  |  | Glutamyltranspeptidase: | | | | |
|  |  |  | Others | | | | |
| Physical sign | Blood pressure（mmHg） | |  | | | | |
|  | Weight（kg） | |  | | | | |
|  | [Height](javascript:void(0);) (cm) | |  | | | | |
|  | Body mass index （BMI） | |  | | | | |
|  | Others | |  | | | | |
| Lifestyles | Daily consumption of cigarettes | |  | | | | |
|  | Daily consumption of  alcohol | |  | | | | |
|  | Others (including food habits and other drugs) | |  | | | | |
|  | Fitness habits | | Form: Time: | | | | |
|  | Dietaryhabits | Yoghurt |  | | | | |
|  |  | staple |  | | | | |
|  | Psychological quality | | 1.better 2.good 3.worse □ | | | | |
|  | Treatment compliance | | 1.better 2.good 3.worse □ | | | | |
| Drugs | Antibiotics | |  | | | | |
|  | Probiotics | |  | | | | |
|  | immunosuppressors | |  | | | | |
|  | Or others | |  | | | | |
|  | Drugs 1: (name) | |  | | | | |
|  | Drug dosage | | Times/Day: Dosage/time: mg | | | |  |
|  | Drugs 2:(name) | |  |  | |  | |
|  | Drug dosage | | Times/Day: Dosage/time: mg | | | |  |
|  | Drugs 3:(name) | |  | | | | |
|  | Drug dosage | | Times/Day: Dosage/time: mg | | | |  |
|  | Others | |  | | | | |
| Other need to supplement | | |  | | | | |
| Patients signature | | |  | | | | |
| Doctor signature | | |  | | | | |

**SUPPLEMENTARY TABLE S2** Detailed data for linear discriminant analysis (LDA) of the fecal microbial OTUs between liver recipients in Group LT_A, LT_N and HCs.

**SUPPLEMENTARY FIGURE S1** Phylogenetic diversity of Fecal microbiomes among individuals and between recipients in Group LT_A, LT_N and healthy subjects. (**A**) Rarefaction analysis of bacterial 16S rRNA gene sequences of each fecal sample to evaluate whether further sequencing would likely detect additional taxa, indicated by a plateau; (**B**) r-shannon index curves, the vertical axis shows that values of r-shannon index that would be expected to be found after sampling the number of reads shown on the horizontal axis; (**C**) Richness index curves that evaluate the number of samples likely required to identify additional taxa indicated by a plateau; (**D**) A venn diagram illustrating overlap of OTUs for the fecal microbiomes of the three groups. A total of 588 OTUs were detected, only78.2% OTUs were detected in all three groups.


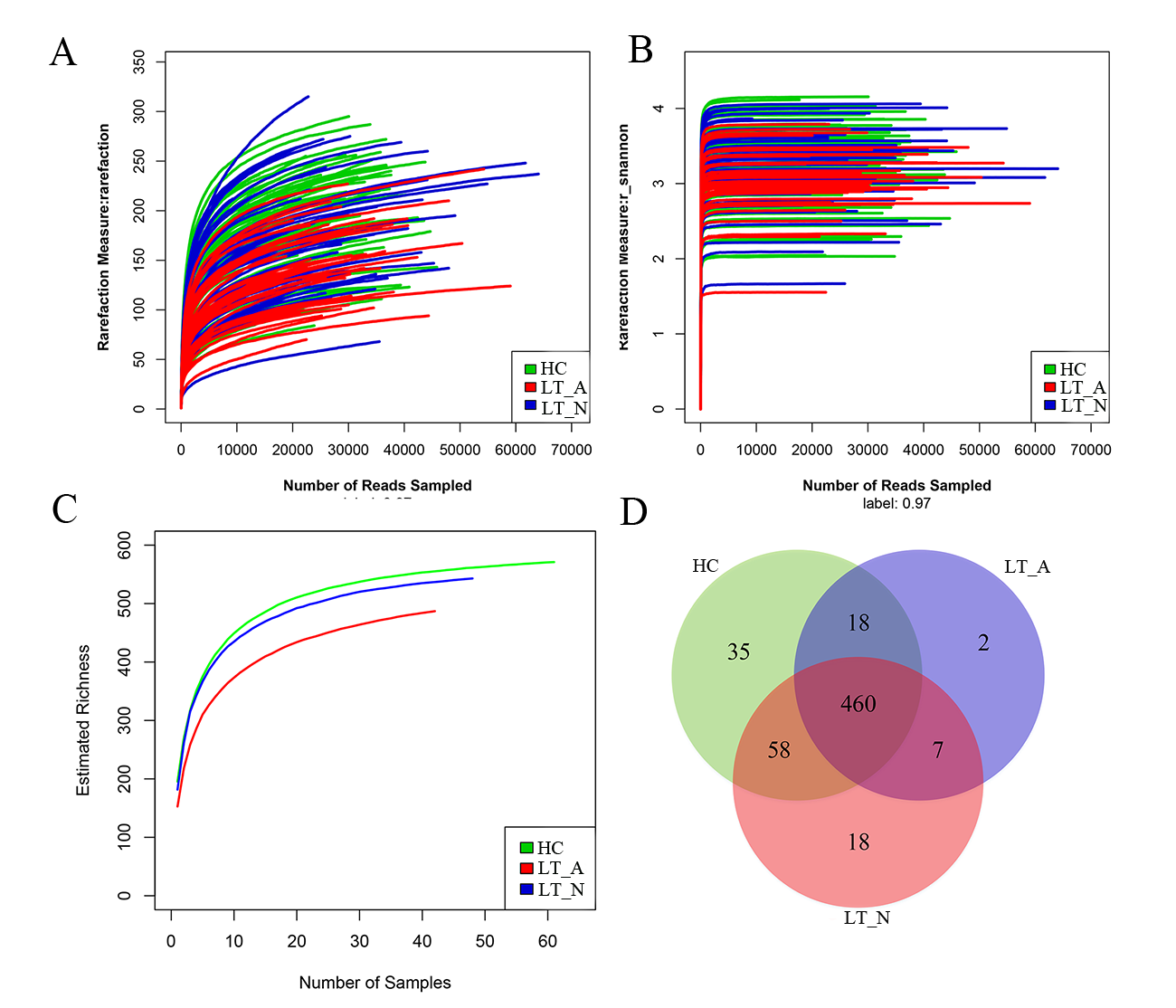


**SUPPLEMENTARY FIGURE S2** Signiﬁcant differences in the relative abundance of predominant class (A) and phylum (B) between the LT patient and the HC groups. Box parameters, the short horizontal solid lines represents median value for each group, and the upper and lower rangers of the box represent the 75% and 25% quartiles, respectively; P-values were calculated using the non-parametric Kruskal–wallis test and Signiﬁcant differences are indicated by P<0.05. LT_N, fecal microbiomes of the liver recipients with normal liver function; LT_A, fecal microbiomes of the liver recipients with abnormal liver function; HC, fecal microbiomes of the healthy control group. The subject group is indicated by the color key at the top right corner.


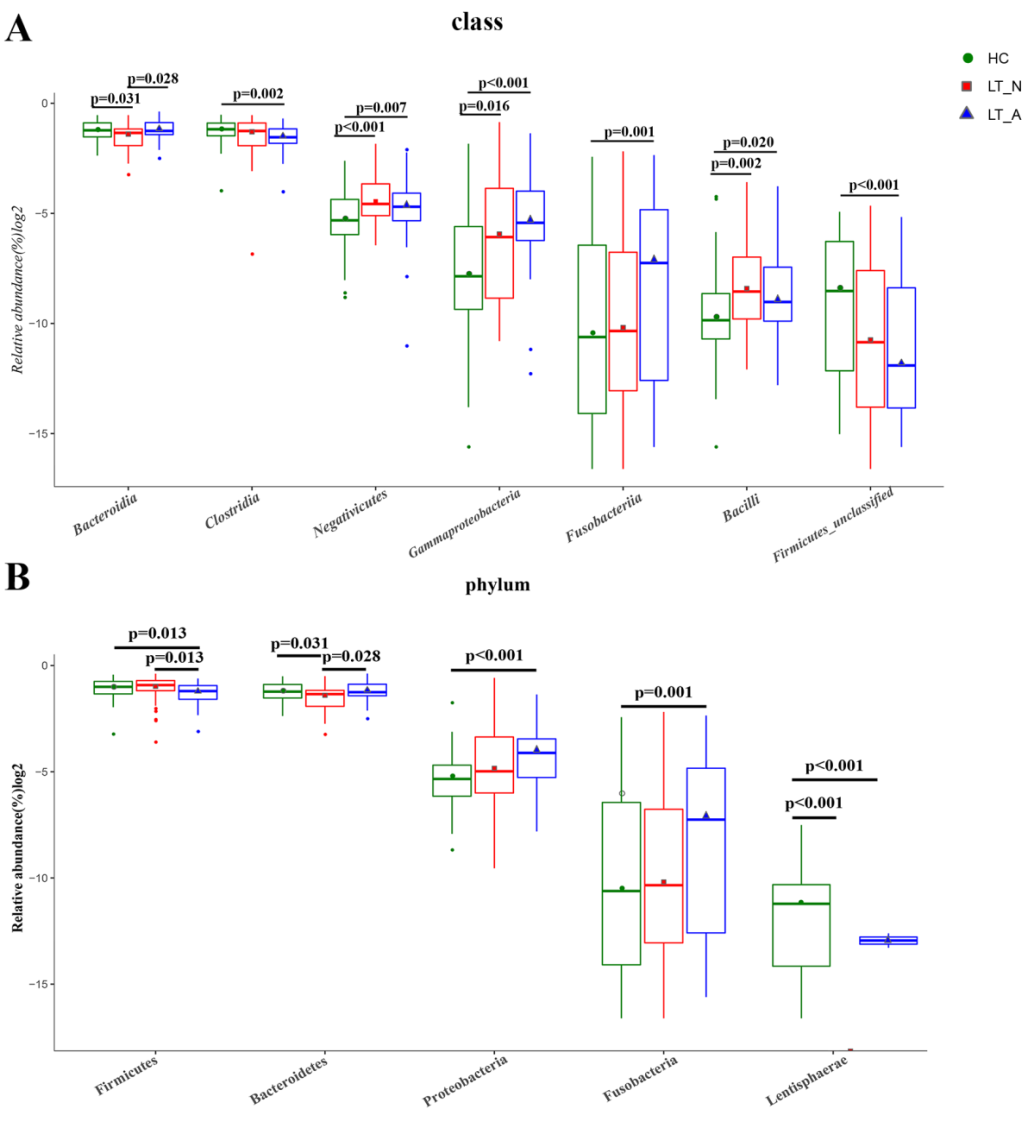


**SUPPLEMENTARY FIGURE S3** Heat maps showing the relative abundance of the discriminatory OTUs that drive the differences between groups LT_N and HC. For each sample, the columns show the relative abundance data for the discriminatory OTUs listed to the right of the figure. The abundance values for each of the genera were clustered using unsupervised hierarchical clustering (the relative abundance of each genus is indicated by a gradient of color from blue (low abundance) to red (high abundance)). The corresponding genus of each key OTU is noted to the right of the figure, and the heat map on the left shows Spearman hierarchical clustering of relative abundance values for each of the 29 most discriminatory 97%-identity OTUs in a random forest-based model of the fecal microbiota of groups LT_N and HC. LT_N, fecal microbiomes of the liver recipients with normal liver function; HC, fecal microbiomes of the healthy control group; OTU, operational taxonomic unit.


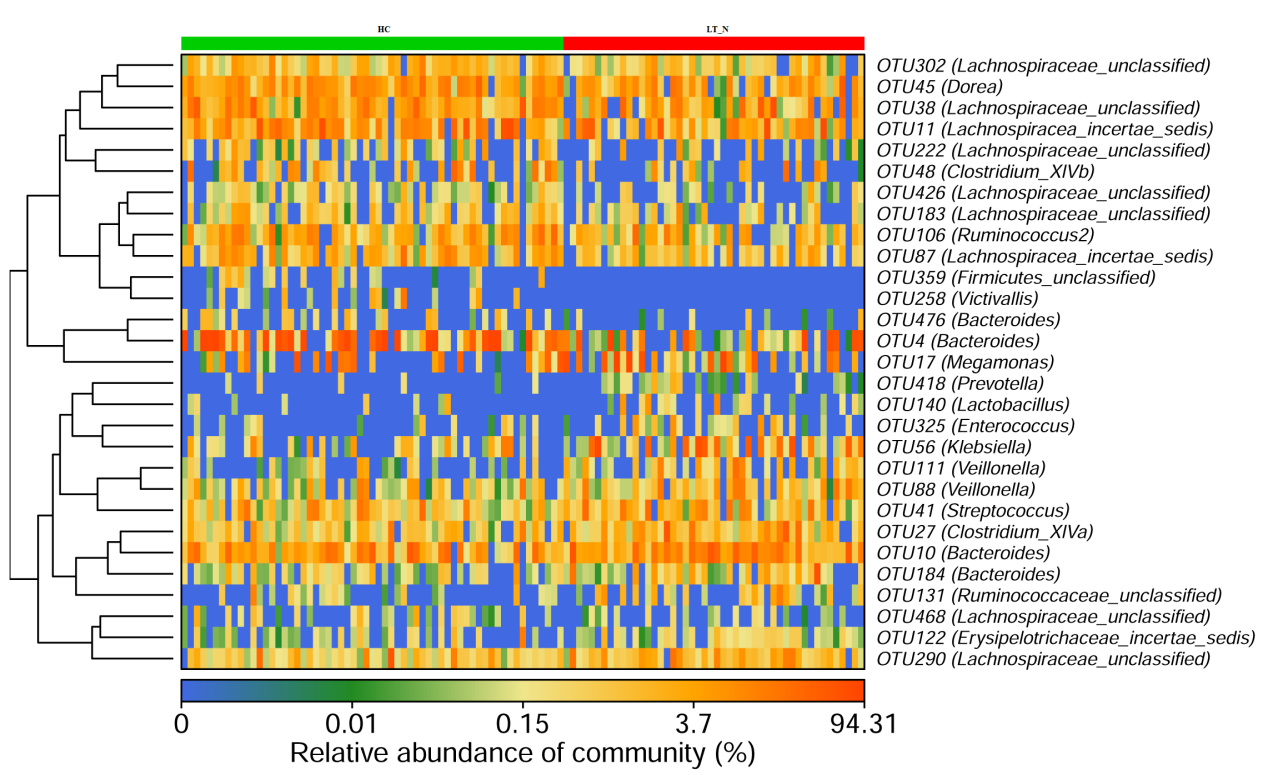


SUPPLEMENTARY FIGURE S4 Spearman's Correlation between the relative abundance of fecal bacterial genera and serum markers of liver dysfunction. Circle sizes and color intensity represent the magnitude of correlation (Red circles, positive correlations; green circles, negative correlations). The red and light blue solid lines indicate a significant correlation at the *p*≤0.01 level, while the red and light blue broken lines indicate a significant correlation at the 0.01<*p*≤0.05 level (the red lines, positive correlations; the light blue lines, negative correlations). ALT, alanine aminotransferase; AST, aspartate aminotransferase; GGT, glutamyl transpeptidase; TB, Total bilirubin.


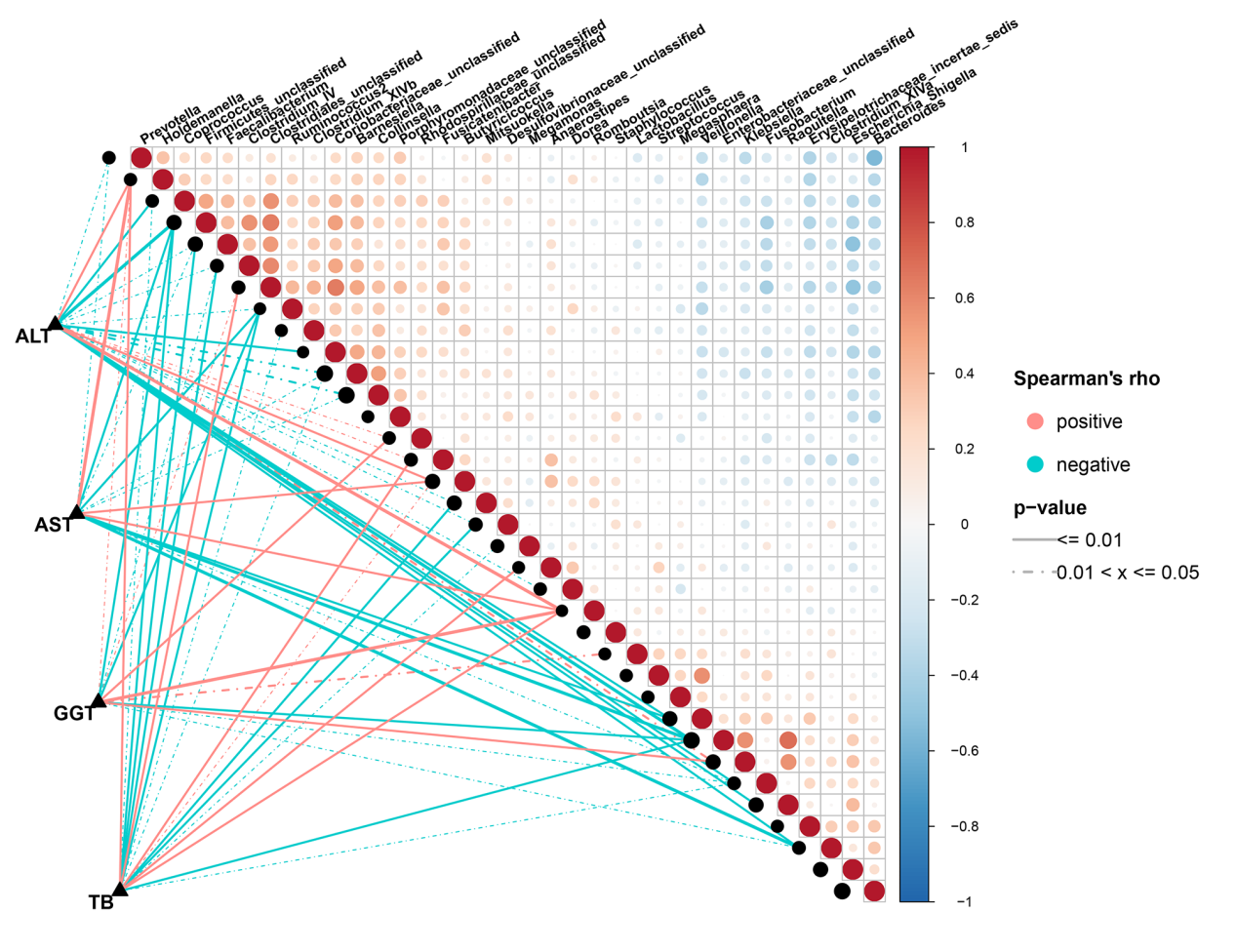


**SUPPLEMENTARY DATASET S1** Sequences determined using the MiSeq System (Illumina). **SUPPLEMENTARY DATASET S1_ A** Annotation of all OTU which detected by 16s rDNA sequencing; **SUPPLEMENTARY DATASET S1_ B** The species-level OTUs and species richness and diversity estimates were obtained for each microbiome.

**SUPPLEMENTARY DATASET S2** Detailed data for discriminatory genera/clusters that drive the differences between Group LT_A, LT_N and HC. **SUPPLEMENTARY DATASET S2_A** Detailed data for the top 20 OTUs and ROC -plot AUC values of Microbial index that drive the differences between LT_A and HC; **SUPPLEMENTARY DATASET S2_B** Detailed data for the top 20 OTUs and ROC -plot AUC values of Microbial index that drive the differences between LT_A and LT_N; **SUPPLEMENTARY DATASET S2_C** Detailed data for the top 20 OTUs and ROC -plot AUC values of Microbial index that drive the differences between LT_N and HC.

**The detailed script of microbial marker identification**

<https://github.com/Neal050617/RFCV>

[**RFCV**](https://github.com/Neal050617/RFCV)/**RFCV2ROC.pl**

| if(@ARGV<5){ |
| --- |
| print "RandomForeat2ROC.pl otu_table feature_importance_file Wilcox_file group top_number\n"; |
| print "The otu_table should be the subset table, contain two group samples\n"; |
| exit; |
| } |
| my $otu_table = shift; |
| my $feature_File = shift; |
| my $Wilcox = shift; |
| my $group = shift; |
| my $top_number = shift; |
|  |
| open(OTU,"<$otu_table") or die; |
| my $head = <OTU>; |
| chomp $head; |
| my %abundance; |
| my @headers = split /\t/,$head; |
| while(<OTU>){ |
| chomp $_; |
| my @line = split /\t/,$_; |
| for(my $i=1;$i<@line;$i++){ |
| ${$abundance{$line[0]}}{$headers[$i]} = $line[$i]; |
| } |
| } |
| close(OTU); |
|  |
| `sed 's/\"//g' $Wilcox >wilcox.tmp.xls`; |
| open(WILCOX,"<wilcox.tmp.xls") or die; |
| my $head_w = <WILCOX>; |
| chomp $head_w; |
| my @headers_w = split /\t/,$head_w; |
| $headers_w[1] =~ /**\(**(**\S**+)**\)**/; |
| $headers_w[1] = $1; |
| #print "$headers_w[1]\n"; |
| $headers_w[3] =~ /**\(**(**\S**+)**\)**/; |
| $headers_w[3] = $1; |
| my @G1; my @G2; |
| my %abundance_G1; my %abundance_G2; |
| while(<WILCOX>){ |
| chomp $_; |
| my @line = split /\t/,$_; |
| $abundance_G1{$line[0]} = $line[1]; |
| $abundance_G2{$line[0]} = $line[3]; |
| #print "$line[0]\tccc\t$line[1]\t$line[3]\t$_\n"; |
| if($line[1] > $line[3]){ |
| #print "$line[0]\t$line[1]\t$line[3]\n"; |
| if(@G1 <= $top_number){ |
| $G1[@G1] = $line[0]; |
| } |
| }else{ |
| if(@G2 <= $top_number){ |
| $G2[@G2] = $line[0]; |
| } |
| } |
| } |
| close(WILCOX); |
|  |
|  |
| my %groups; |
| open(GROUP,"<$group") or die; |
| while(<GROUP>){ |
| chomp $_; |
| my @line = split /\t/,$_; |
| $groups{$line[0]} = $line[1]; |
| } |
| close(GROUP); |
|  |
| my @RF1; my @RF2; |
| my $mark_index=0; |
| open(RF,"<$feature_File") or die; |
| <RF>; |
| while(<RF>){ |
| chomp $_; |
| $mark_index++; |
| my @line = split /\t/,$_; |
| if($mark_index<= $top_number){ |
| if($abundance_G1{$line[0]} >$abundance_G2{$line[0]}){ |
| #if(@RF1 < $top_number){ |
| $RF1[@RF1] = $line[0]; |
| #} |
| }else{ |
| #if(@RF2 < $top_number){ |
| $RF2[@RF2] = $line[0]; |
| #} |
| } |
| } |
| } |
| close(RF); |
|  |
| my $aaa = join("\t",@RF1); |
| my $bbb = join("\t",@RF2); |
| open(COMB,">combination.details.xls") or die; |
| open(ROC,">ROC.combination.xls") or die; |
| print COMB "$headers_w[1] Group top $top_number OTUs: $aaa\n"; |
| print COMB "$headers_w[3] Group top $top_number OTUs: $bbb\n"; |
| print COMB "Combination\t$headers_w[1] richness\t$headers_w[3] richness\n"; |
| my $tmp = join("\t",@headers); |
| print ROC "$tmp\n"; |
| print ROC "GROUP\t"; |
| my @Mark; |
| for($i=1;$i<@headers;$i++){ |
| $mark[@mark] = $groups{$headers[$i]}; |
| } |
| my $tmp2 = join("\t",@mark); |
| print ROC "$tmp2\n"; |
|  |
| my @details_RF1; my @details_RF2; |
| for($m=0;$m<=@RF1;$m++){ |
| for($n=0;$n<=@RF2;$n++){ |
| if($m+$n>0){ |
| my $tmp3 = "MIA_".$headers_w[1].$m.$headers_w[3].$n; |
| print ROC "$tmp3\t"; |
| print COMB "$tmp3:\t"; |
| my @combb1; |
| my @abund_tmp1; my @abund_comb1; |
| if($m>0){ |
| for($p=0;$p<=$m-1;$p++){ |
| $combb1[@combb1] = $RF1[$p]; |
| for($i=1;$i<@headers;$i++){ |
| $abund_tmp1[$i-1] += ${$abundance{$RF1[$p]}}{$headers[$i]}; |
| #$combb1[@combb1] = $RF1[$p]; |
| } |
| } |
| $p--; |
| for($i=1;$i<@headers;$i++){ |
| $abund_comb1[$i-1] = $abund_tmp1[$i-1]/($m-$p); |
| } |
| }else{ |
| for($i=1;$i<@headers;$i++){ |
| $abund_comb1[$i-1] = 0; |
| $combb1[0] ="None"; |
| } |
| } |
| my @abund_tmp2; my @abund_comb2; |
| my @combb2; |
| if($n>0){ |
| for($q=0;$q<=$n-1;$q++){ |
| $combb2[@combb2] = $RF2[$q]; |
| for($i=1;$i<@headers;$i++){ |
| $abund_tmp2[$i-1] += ${$abundance{$RF2[$q]}}{$headers[$i]}; |
| #$combb2[@combb2] = $RF2[$q]; |
| } |
| } |
| $q--; |
| for($i=1;$i<@headers;$i++){ |
| $abund_comb2[$i-1] = $abund_tmp2[$i-1]/($n-$q); |
| } |
| }else{ |
| for($i=1;$i<@headers;$i++){ |
| $abund_comb2[$i-1] = 0; |
| $combb2[0] ="None"; |
| } |
| } |
| my @abund_MIA; |
| for($i=1;$i<@headers;$i++){ |
| $abund_MIA[$i-1] = $abund_comb1[$i-1] - $abund_comb2[$i-1]; |
| } |
| my $tmp_RF = join("\t",@abund_MIA); |
| print ROC "$tmp_RF\n"; |
| my $tmp_comb1 = join(";",@combb1); |
| my $tmp_comb2 = join(";",@combb2); |
| print COMB "$tmp_comb1\t$tmp_comb2\n"; |
| } |
| } |
| } |
|  |
| `more ROC.combination.xls \|awk -F"\t" '{for(i=1;i<=NF;i++){a[FNR,i]=\$i}}END{for(i=1;i<=NF;i++){for(j=1;j<=FNR;j++){printf a[j,i]"\t"}print ""}}' >ROC.combination.T.xls`; |
| open(RCMD,">cmd.r") or die; |
| print RCMD " |
| library(pROC) |
| data <-read.table(file=\"ROC.combination.T.xls\",header=T,check.names=FALSE,sep=\"\\t\") |
| comb <-dim(data)[2]-1 |
| a<-c(rep(\"NA\",comb-2)) |
| b<-c(rep(0,comb-2)) |
| for(i in 3:comb){ |
| name <- colnames(data)[i] |
| pdf(paste(name,\".pdf\",sep=\"\")) |
| roc <- roc(data[,2],data[,i],plot=T,col=\"red\",print.thres=T,print.auc=T) |
| dev.off() |
| a[i-2] <- colnames(data)[i] |
| b[i-2] <- roc\$auc |
| c<- data.frame(a,b) |
| } |
| colnames(c) <- c(\"Combation\",\"ROC_AUC\") |
| write.table(c,\"ROC.combination.AUC.xls\",sep=\"\t\") |
| "; |
| `R --restore --no-save < cmd.r`; |

| @@ -0,0 +1,787 @@ | |
| --- | --- |
|  | qvalue <- function(p=NULL, lambda=seq(0,0.90,0.05), pi0.method="smoother", fdr.level=NULL, robust=FALSE, |
|  | gui=FALSE, smooth.df = 3, smooth.log.pi0 = FALSE) { |
|  | #Input |
|  | #============================================================================= |
|  | #p: a vector of p-values (only necessary input) |
|  | #fdr.level: a level at which to control the FDR (optional) |
|  | #lambda: the value of the tuning parameter to estimate pi0 (optional) |
|  | #pi0.method: either "smoother" or "bootstrap"; the method for automatically |
|  | # choosing tuning parameter in the estimation of pi0, the proportion |
|  | # of true null hypotheses |
|  | #robust: an indicator of whether it is desired to make the estimate more robust |
|  | # for small p-values and a direct finite sample estimate of pFDR (optional) |
|  | #gui: A flag to indicate to 'qvalue' that it should communicate with the gui. ## change by Alan |
|  | # Should not be specified on command line. |
|  | #smooth.df: degrees of freedom to use in smoother (optional) |
|  | #smooth.log.pi0: should smoothing be done on log scale? (optional) |
|  | # |
|  | #Output |
|  | #============================================================================= |
|  | #call: gives the function call |
|  | #pi0: an estimate of the proportion of null p-values |
|  | #qvalues: a vector of the estimated q-values (the main quantity of interest) |
|  | #pvalues: a vector of the original p-values |
|  | #significant: if fdr.level is specified, an indicator of whether the q-value |
|  | # fell below fdr.level (taking all such q-values to be significant controls |
|  | # FDR at level fdr.level) |
|  |  |
|  | #Set up communication with GUI, if appropriate |
|  | # print(sys.calls()) |
|  | # print(sys.frames()) |
|  |  |
|  | # if(gui) { |
|  | # idx <- (1:sys.nframe())[as.character(sys.calls()) == "qvalue.gui()"] |
|  | # gui.env <- sys.frames()[[idx]] |
|  | # } |
|  |  |
|  | #This is just some pre-processing |
|  | if(is.null(p)) ## change by Alan |
|  | {qvalue.gui(); return("Launching point-and-click...")} |
|  | if(gui & !interactive()) ## change by Alan |
|  | gui = FALSE |
|  |  |
|  | if(min(p)<0 \|\| max(p)>1) { |
|  | if(gui) ## change by Alan: check for GUI |
|  | eval(expression(postMsg(paste("ERROR: p-values not in valid range.", "\n"))), parent.frame()) |
|  | else |
|  | print("ERROR: p-values not in valid range.") |
|  | return(0) |
|  | } |
|  | if(length(lambda)>1 && length(lambda)<4) { |
|  | if(gui) |
|  | eval(expression(postMsg(paste("ERROR: If length of lambda greater than 1, you need at least 4 values.", |
|  | "\n"))), parent.frame()) |
|  | else |
|  | print("ERROR: If length of lambda greater than 1, you need at least 4 values.") |
|  | return(0) |
|  | } |
|  | if(length(lambda)>1 && (min(lambda) < 0 \|\| max(lambda) >= 1)) { ## change by Alan: check for valid range for lambda |
|  | if(gui) |
|  | eval(expression(postMsg(paste("ERROR: Lambda must be within [0, 1).", "\n"))), parent.frame()) |
|  | else |
|  | print("ERROR: Lambda must be within [0, 1).") |
|  | return(0) |
|  | } |
|  | m <- length(p) |
|  | #These next few functions are the various ways to estimate pi0 |
|  | if(length(lambda)==1) { |
|  | if(lambda<0 \|\| lambda>=1) { ## change by Alan: check for valid range for lambda |
|  | if(gui) |
|  | eval(expression(postMsg(paste("ERROR: Lambda must be within [0, 1).", "\n"))), parent.frame()) |
|  | else |
|  | print("ERROR: Lambda must be within [0, 1).") |
|  | return(0) |
|  | } |
|  |  |
|  | pi0 <- mean(p >= lambda)/(1-lambda) |
|  | pi0 <- min(pi0,1) |
|  | } |
|  | else { |
|  | pi0 <- rep(0,length(lambda)) |
|  | for(i in 1:length(lambda)) { |
|  | pi0[i] <- mean(p >= lambda[i])/(1-lambda[i]) |
|  | } |
|  |  |
|  | if(pi0.method=="smoother") { |
|  | if(smooth.log.pi0) |
|  | pi0 <- log(pi0) |
|  |  |
|  | spi0 <- smooth.spline(lambda,pi0,df=smooth.df) |
|  | pi0 <- predict(spi0,x=max(lambda))$y |
|  |  |
|  | if(smooth.log.pi0) |
|  | pi0 <- exp(pi0) |
|  | pi0 <- min(pi0,1) |
|  | } |
|  | else if(pi0.method=="bootstrap") { |
|  | minpi0 <- min(pi0) |
|  | mse <- rep(0,length(lambda)) |
|  | pi0.boot <- rep(0,length(lambda)) |
|  | for(i in 1:100) { |
|  | p.boot <- sample(p,size=m,replace=TRUE) |
|  | for(i in 1:length(lambda)) { |
|  | pi0.boot[i] <- mean(p.boot>lambda[i])/(1-lambda[i]) |
|  | } |
|  | mse <- mse + (pi0.boot-minpi0)^2 |
|  | } |
|  | pi0 <- min(pi0[mse==min(mse)]) |
|  | pi0 <- min(pi0,1) |
|  | } |
|  | else { ## change by Alan: check for valid choice of 'pi0.method' (only necessary on command line) |
|  | print("ERROR: 'pi0.method' must be one of 'smoother' or 'bootstrap'.") |
|  | return(0) |
|  | } |
|  | } |
|  | if(pi0 <= 0) { |
|  | if(gui) |
|  | eval(expression(postMsg( |
|  | paste("ERROR: The estimated pi0 <= 0. Check that you have valid p-values or use another lambda method.", |
|  | "\n"))), parent.frame()) |
|  | else |
|  | print("ERROR: The estimated pi0 <= 0. Check that you have valid p-values or use another lambda method.") |
|  | return(0) |
|  | } |
|  | if(!is.null(fdr.level) && (fdr.level<=0 \|\| fdr.level>1)) { ## change by Alan: check for valid fdr.level |
|  | if(gui) |
|  | eval(expression(postMsg(paste("ERROR: 'fdr.level' must be within (0, 1].", "\n"))), parent.frame()) |
|  | else |
|  | print("ERROR: 'fdr.level' must be within (0, 1].") |
|  | return(0) |
|  | } |
|  | #The estimated q-values calculated here |
|  | u <- order(p) |
|  |  |
|  | # change by Alan |
|  | # ranking function which returns number of observations less than or equal |
|  | qvalue.rank <- function(x) { |
|  | idx <- sort.list(x) |
|  |  |
|  | fc <- factor(x) |
|  | nl <- length(levels(fc)) |
|  | bin <- as.integer(fc) |
|  | tbl <- tabulate(bin) |
|  | cs <- cumsum(tbl) |
|  |  |
|  | tbl <- rep(cs, tbl) |
|  | tbl[idx] <- tbl |
|  |  |
|  | return(tbl) |
|  | } |
|  |  |
|  | v <- qvalue.rank(p) |
|  |  |
|  | qvalue <- pi0*m*p/v |
|  | if(robust) { |
|  | qvalue <- pi0*m*p/(v*(1-(1-p)^m)) |
|  | } |
|  | qvalue[u[m]] <- min(qvalue[u[m]],1) |
|  | for(i in (m-1):1) { |
|  | qvalue[u[i]] <- min(qvalue[u[i]],qvalue[u[i+1]],1) |
|  | } |
|  | #The results are returned |
|  | if(!is.null(fdr.level)) { |
|  | retval <- list(call=match.call(), pi0=pi0, qvalues=qvalue, pvalues=p, fdr.level=fdr.level, ## change by Alan |
|  | significant=(qvalue <= fdr.level), lambda=lambda) |
|  | } |
|  | else { |
|  | retval <- list(call=match.call(), pi0=pi0, qvalues=qvalue, pvalues=p, lambda=lambda) |
|  | } |
|  | class(retval) <- "qvalue" |
|  | return(retval) |
|  | } |
|  |  |
|  | qplot <- function(qobj, rng=c(0.0, 0.1), smooth.df = 3, smooth.log.pi0 = FALSE, ...) { ## change by Alan: |
|  | ## 'rng' a vector instead of an upper bound alone |
|  | #Input |
|  | #============================================================================= |
|  | #qobj: a q-value object returned by the qvalue function |
|  | #rng: the range of q-values to be plotted (optional) |
|  | #smooth.df: degrees of freedom to use in smoother (optional) |
|  | #smooth.log.pi0: should smoothing be done on log scale? (optional) |
|  | # |
|  | #Output |
|  | #============================================================================= |
|  | #Four plots: |
|  | #Upper-left: pi0.hat(lambda) versus lambda with a smoother |
|  | #Upper-right: q-values versus p-values |
|  | #Lower-left: number of significant tests per each q-value cut-off |
|  | #Lower-right: number of expected false positives versus number of significant tests |
|  | q2 <- qobj$qval[order(qobj$pval)] |
|  | if(min(q2) > rng[2]) {rng <- c(min(q2), quantile(q2, 0.1))} ## change by Alan: replace 'rng' with vector |
|  | p2 <- qobj$pval[order(qobj$pval)] |
|  | par(mfrow=c(2,2)) |
|  | lambda <- qobj$lambda |
|  | if(length(lambda)==1) {lambda <- seq(0,max(0.90,lambda),0.05)} |
|  | pi0 <- rep(0,length(lambda)) |
|  | for(i in 1:length(lambda)) { |
|  | pi0[i] <- mean(p2>lambda[i])/(1-lambda[i]) |
|  | } |
|  |  |
|  | if(smooth.log.pi0) |
|  | pi0 <- log(pi0) |
|  | spi0 <- smooth.spline(lambda,pi0,df=smooth.df) |
|  |  |
|  | if(smooth.log.pi0) { |
|  | pi0 <- exp(pi0) |
|  | spi0$y <- exp(spi0$y) |
|  | } |
|  |  |
|  | pi00 <- round(qobj$pi0,3) |
|  | plot(lambda,pi0,xlab=expression(lambda),ylab=expression(hat(pi)[0](lambda)),pch=".") |
|  | mtext(substitute(hat(pi)[0] == that, list(that= pi00))) |
|  | lines(spi0) |
|  |  |
|  | plot(p2[q2 >= rng[1] & q2 <= rng[2]], q2[q2 >= rng[1] & q2 <= rng[2]], type = "l", xlab = "p-value", ## changes by Alan |
|  | ylab = "q-value") |
|  | plot(q2[q2 >= rng[1] & q2 <= rng[2]], (1 + sum(q2 < rng[1])):sum(q2 <= rng[2]), type="l", |
|  | xlab="q-value cut-off", ylab="significant tests") |
|  | plot((1 + sum(q2 < rng[1])):sum(q2 <= rng[2]), q2[q2 >= rng[1] & q2 <= rng[2]] * |
|  | (1 + sum(q2 < rng[1])):sum(q2 <= rng[2]), type = "l", xlab = "significant tests", |
|  | ylab = "expected false positives") |
|  | par(mfrow=c(1,1)) |
|  | } |
|  |  |
|  | plot.qvalue <- function(x, ...) qplot(x, ...) |
|  |  |
|  | qwrite <- function(qobj, filename="my-qvalue-results.txt") { |
|  | #Input |
|  | #============================================================================= |
|  | #qobj: a q-value object returned by the qvalue function |
|  | #filename: the name of the file where the results are written |
|  | # |
|  | #Output |
|  | #============================================================================= |
|  | #A file sent to "filename" with the following: |
|  | #First row: the estimate of the proportion of true negatives, pi0 |
|  | #Second row: FDR significance level (if specified) ## change by Alan |
|  | #Third row and below: the p-values (1st column), the estimated q-values (2nd column), |
|  | # and indicator of significance level if appropriate (3rd column) |
|  | cat(c("pi0:", qobj$pi0, "\n\n"), file=filename, append=FALSE) |
|  | if(any(names(qobj) == "fdr.level")) { |
|  | cat(c("FDR level:", qobj$fdr.level, "\n\n"), file=filename, append=TRUE) |
|  | cat(c("p-value q-value significant", "\n"), file=filename, append=TRUE) ## change by Alan (space-delimited now) |
|  | # for(i in 1:length(qobj$qval)) { |
|  | # cat(c(qobj$pval[i], "\t", qobj$qval[i], "\t", qobj$significant[i], "\n"), file=filename, append=TRUE) |
|  | # } |
|  | write(t(cbind(qobj$pval, qobj$qval, qobj$significant)), file=filename, ncolumns=3, append=TRUE) ## change by Alan |
|  | } |
|  | else { |
|  | cat(c("p-value q-value", "\n"), file=filename, append=TRUE) |
|  | # for(i in 1:length(qobj$qval)) { |
|  | # cat(c(qobj$pval[i], "\t", qobj$qval[i], "\n"), file=filename, append=TRUE) |
|  | # } |
|  | write(t(cbind(qobj$pval, qobj$qval)), file=filename, ncolumns=2, append=TRUE) |
|  | } |
|  | } |
|  |  |
|  | qsummary <- function (qobj, cuts=c(0.0001, 0.001, 0.01, 0.025, 0.05, 0.10, 1), digits=getOption("digits"), ...) { |
|  | cat("\nCall:\n", deparse(qobj$call), "\n\n", sep = "") |
|  | cat("pi0:",format(qobj$pi0, digits=digits),"\n", sep="\t") |
|  | cat("\n") |
|  | cat("Cumulative number of significant calls:\n") |
|  | cat("\n") |
|  | counts <- sapply(cuts, function(x) c("p-value"=sum(qobj$pvalues < x), "q-value"=sum(qobj$qvalues < x))) |
|  | colnames(counts) <- paste("<", cuts, sep="") |
|  | print(counts) |
|  | cat("\n") |
|  | invisible(qobj) |
|  | } |
|  |  |
|  | summary.qvalue <- function(object, ...) { |
|  | qsummary(object, ...) |
|  | } |
|  |  |
|  | #################################################### |
|  | ## TCL-TK GUI for John Storey's Q-Value Software. ## |
|  | ## Alan Dabney, 10/01/03 ## |
|  | #################################################### |
|  |  |
|  | qvalue.gui <- function(dummy = NULL) { |
|  |  |
|  | if(interactive()) { |
|  |  |
|  | out <- NULL |
|  | inFileName.var <- tclVar("") |
|  | pp <- NULL |
|  | from.var.1 = tclVar("0.0") |
|  | to.var.1 = tclVar("0.90") |
|  | by.var.1 = tclVar("0.05") |
|  | from.var.2 = tclVar("0.0") |
|  | to.var.2 = tclVar("0.1") |
|  | single.var = tclVar("") |
|  | lambda.var = tclVar(1) |
|  | pi0.var = tclVar(1) |
|  | df.var = tclVar("3") |
|  | log.no.var = tclVar(1) |
|  |  |
|  | robust.var = tclVar(0) |
|  | levelSpec.var = tclVar(0) |
|  | level.var = tclVar("0.05") |
|  | plotChoice.var = tclVar(1) |
|  |  |
|  | titleFont <- "Helvetica 14" |
|  | normalFont <- "Helvetica 10" |
|  |  |
|  | ######################## |
|  | ## Utility functions ## |
|  | ######################## |
|  |  |
|  | findPVals <- function() { |
|  | tclvalue(inFileName.var) <- tclvalue(tkgetOpenFile()) |
|  | } |
|  |  |
|  | readPVals <- function() { |
|  | flnm <- tclvalue(inFileName.var) |
|  |  |
|  | if(flnm == "") { |
|  | postMsg("ERROR: No file selected.\n") |
|  | } |
|  |  |
|  | else { |
|  | postMsg("Reading p-values...") |
|  | pvals = scan(flnm) |
|  | if(is.null(pvals) == FALSE) { |
|  | assign("pp", pvals, inherits = TRUE) |
|  | postMsg("done.\n") |
|  | } |
|  | } |
|  | } |
|  |  |
|  | lambda.fnc <- function() { |
|  | if(tclvalue(lambda.var) == 1) { |
|  | tkconfigure(from.ety.1, state = "normal") |
|  | tkconfigure(to.ety.1, state = "normal") |
|  | tkconfigure(by.ety.1, state = "normal") |
|  | tkconfigure(single.ety, state = "disabled") |
|  | } |
|  | else { |
|  | tkconfigure(from.ety.1, state = "disabled") |
|  | tkconfigure(to.ety.1, state = "disabled") |
|  | tkconfigure(by.ety.1, state = "disabled") |
|  | tkconfigure(single.ety, state = "normal") |
|  | } |
|  | } |
|  |  |
|  | smoother.fnc <- function() { |
|  | if(tclvalue(pi0.var) == 1) |
|  | tkconfigure(smoothOptions.btn, state = "normal") |
|  | else |
|  | tkconfigure(smoothOptions.btn, state = "disabled") |
|  | } |
|  |  |
|  | smoothOptions.fnc <- function() { |
|  | base <- tktoplevel() |
|  | tkwm.title(base, "Smoother") |
|  |  |
|  | df.var.0 <- tclvalue(df.var) |
|  | log.no.var.0 <- tclvalue(log.no.var) |
|  |  |
|  | smooth.ok.fnc <- function() { |
|  | tkdestroy(base) |
|  | } |
|  |  |
|  | smooth.cancel.fnc <- function() { |
|  | tclvalue(df.var) <- df.var.0 |
|  | tclvalue(log.no.var) <- log.no.var.0 |
|  |  |
|  | tkdestroy(base) |
|  | } |
|  |  |
|  | top.frm <- tkframe(base, borderwidth = 2) |
|  | inset.frm <- tkframe(top.frm, relief = "raised", bd = 2) |
|  |  |
|  | df.frm <- tkframe(inset.frm) |
|  | df.lbl <- tklabel(df.frm, text = "Degrees of freedom:", font = normalFont) |
|  | df.ety <- tkentry(df.frm, textvariable = df.var, font = normalFont, width = 3, justify = "center") |
|  | tkpack(df.lbl, side = "left") |
|  | tkpack(df.ety, side = "left") |
|  |  |
|  | log.lbl.frm <- tkframe(inset.frm) |
|  | log.lbl <- tklabel(log.lbl.frm, text = "Variable to smooth:", font = normalFont) |
|  | tkpack(log.lbl, side = "left") |
|  |  |
|  | log.no.frm <- tkframe(inset.frm) |
|  | log.no.cbtn <- tkradiobutton(log.no.frm, text = "pi0", font = normalFont, |
|  | variable = log.no.var, value = 1) |
|  | tkpack(log.no.cbtn, side = "left") |
|  |  |
|  | log.yes.frm <- tkframe(inset.frm) |
|  | log.yes.cbtn <- tkradiobutton(log.yes.frm, text = "log pi0", font = normalFont, |
|  | variable = log.no.var, value = 0) |
|  | tkpack(log.yes.cbtn, side = "left") |
|  |  |
|  | btn.frm <- tkframe(inset.frm) |
|  | ok.btn <- tkbutton(btn.frm, text = "OK", font = normalFont, command = smooth.ok.fnc) |
|  | cancel.btn <- tkbutton(btn.frm, text = "Cancel", font = normalFont, command = smooth.cancel.fnc) |
|  | tkgrid(ok.btn, cancel.btn) |
|  |  |
|  | tkpack(df.frm, padx = 5, anchor = "w", fill = "x", expand = TRUE) |
|  | tkpack(log.lbl.frm, padx = 5, anchor = "w", fill = "x", expand = TRUE) |
|  | tkpack(log.no.frm, padx = 10, anchor = "w", fill = "x", expand = TRUE) |
|  | tkpack(log.yes.frm, padx = 10, anchor = "w", fill = "x", expand = TRUE) |
|  | tkpack(btn.frm, anchor = "e") |
|  | tkpack(inset.frm) |
|  | tkpack(top.frm) |
|  | } |
|  |  |
|  | level.fnc <- function() { |
|  | if(tclvalue(levelSpec.var) == 1) |
|  | tkconfigure(level.ety, state = "normal") |
|  | else |
|  | tkconfigure(level.ety, state = "disabled") |
|  | } |
|  |  |
|  | execute.fnc <- function() { |
|  | if(is.null(pp)) |
|  | postMsg("ERROR: P-values haven't been read yet.\n") |
|  |  |
|  | else { |
|  | postMsg("Computing q-values...") |
|  | if(tclvalue(lambda.var) == 1) |
|  | lambda <- seq(from = as.numeric(tclvalue(from.var.1)), to = as.numeric(tclvalue(to.var.1)), |
|  | by = as.numeric(tclvalue(by.var.1))) |
|  | else { |
|  | lambda <- as.numeric(tclvalue(single.var)) |
|  | if(is.na(lambda)) { |
|  | postMsg("ERROR: Please specify value for lambda.\n") |
|  | return() |
|  | } |
|  | # else if(lambda <= 0.0 \|\| lambda >= 1.0) { |
|  | # postMsg("ERROR: Lambda must be between 0.0 and 1.0.\n") |
|  | # return() |
|  | # } |
|  | } |
|  | if(tclvalue(pi0.var) == 1) |
|  | pi0.method <- "smoother" |
|  | else |
|  | pi0.method <- "bootstrap" |
|  | if(tclvalue(levelSpec.var) == 1) { |
|  | fdr.level <- as.numeric(tclvalue(level.var)) |
|  | if(is.na(fdr.level)) { |
|  | postMsg("ERROR: Please specify FDR level.\n") |
|  | return() |
|  | } |
|  | # else if(fdr.level <= 0.0 \|\| fdr.level >= 1.0) { |
|  | # postMsg("aborted.\n") |
|  | # postMsg("FDR level must be between 0.0 and 1.0.\n") |
|  | # return() |
|  | # } |
|  | } |
|  | else |
|  | fdr.level <- NULL |
|  | if(tclvalue(robust.var) == 1) |
|  | robust <- TRUE |
|  | else |
|  | robust <- FALSE |
|  | if(tclvalue(log.no.var) == 1) |
|  | smooth.log.pi0 = TRUE |
|  | else |
|  | smooth.log.pi0 = FALSE |
|  |  |
|  | qout = qvalue(p = pp, lambda = lambda, pi0.method = pi0.method, fdr.level = fdr.level, |
|  | robust = robust, gui = TRUE, smooth.df = as.numeric(tclvalue(df.var)), smooth.log.pi0 = smooth.log.pi0) |
|  | if(class(qout) == "qvalue") { |
|  | tclvalue(to.var.2) = as.character(round(qout$pi0, 4)) |
|  | assign("out", qout, inherits = TRUE) |
|  | postMsg(paste("done: pi_0 = ", round(qout$pi0, 4), ".\n", sep = "")) |
|  | } |
|  | } |
|  | } |
|  |  |
|  | plotChoice.fnc <- function() { |
|  | if(tclvalue(plotChoice.var) == 1 \| tclvalue(plotChoice.var) == 2) { |
|  | tkconfigure(from.ety.2, state = "disabled") |
|  | tkconfigure(to.ety.2, state = "disabled") |
|  | } |
|  | else { |
|  | tkconfigure(from.ety.2, state = "normal") |
|  | tkconfigure(to.ety.2, state = "normal") |
|  | } |
|  | } |
|  |  |
|  | histPVals <- function() { |
|  | if(is.null(pp)) |
|  | postMsg("ERROR: P-values haven't been read yet.\n") |
|  |  |
|  | else { |
|  | par(mfrow = c(1, 1)) |
|  | hist(pp, main = "Histogram of P-Values") |
|  | } |
|  | } |
|  |  |
|  |  |
|  | plot.fnc <- function() { |
|  | if(tclvalue(plotChoice.var) == 1) { |
|  | if(is.null(pp)) |
|  | postMsg("ERROR: P-values haven't been read yet.\n") |
|  |  |
|  | else { |
|  | par(mfrow = c(1, 1)) |
|  | hist(pp, main = "Histogram of P-Values", xlab = "") |
|  | } |
|  | } |
|  |  |
|  | else if(tclvalue(plotChoice.var) == 2) { |
|  | if(is.null(out)) |
|  | postMsg("ERROR: Q-values haven't been computed yet.\n") |
|  | else if(class(out) == "qvalue") { |
|  | par(mfrow = c(1, 1)) |
|  | hist(out$qvalues, main = "Histogram of Q-Values", xlab = "") |
|  | } |
|  | } |
|  |  |
|  | else { |
|  | if(tclvalue(log.no.var) == 1) |
|  | smooth.log.pi0 = TRUE |
|  | else |
|  | smooth.log.pi0 = FALSE |
|  |  |
|  | if(is.null(out)) |
|  | postMsg("ERROR: Q-values haven't been computed yet.\n") |
|  | else if(class(out) == "qvalue") |
|  | qplot(out, rng = as.numeric(c(tclvalue(from.var.2), tclvalue(to.var.2))), |
|  | smooth.df = as.numeric(tclvalue(df.var)), smooth.log.pi0 = smooth.log.pi0) |
|  | } |
|  | } |
|  |  |
|  | saveOutput.fnc <- function() { |
|  | if(is.null(out)) |
|  | postMsg("ERROR: Q-values haven't been computed yet.\n") |
|  |  |
|  | else if(class(out) == "qvalue") { |
|  | postMsg("Writing results to file...") |
|  | flnm <- tclvalue(tkgetSaveFile()) |
|  | if(flnm != "") { |
|  | qwrite(out, filename = flnm) |
|  | postMsg("done.\n") |
|  | } |
|  | else |
|  | postMsg("aborted.\n") |
|  | } |
|  | } |
|  |  |
|  | savePlot.fnc <- function() { |
|  | if(tclvalue(plotChoice.var) == 1) { |
|  | if(is.null(pp)) |
|  | postMsg("ERROR: P-values haven't been read yet.\n") |
|  | else { |
|  | flnm <- tclvalue(tkgetSaveFile(defaultextension = "pdf", filetypes = "{{PDF File} {.pdf}}")) |
|  | if(flnm != "") { |
|  | pdf(flnm) |
|  | par(mfrow = c(1, 1)) |
|  | hist(pp, main = "Histogram of P-Values", xlab = "") |
|  | dev.off() |
|  | postMsg("Plot saved.\n") |
|  | } |
|  | else |
|  | postMsg("No file selected. Plot not saved.\n") |
|  | } |
|  | } |
|  |  |
|  | else if(tclvalue(plotChoice.var) == 2) { |
|  | if(is.null(out)) |
|  | postMsg("ERROR: Q-values haven't been computed yet.\n") |
|  | else if(class(out) == "qvalue") { |
|  | flnm <- tclvalue(tkgetSaveFile(defaultextension = "pdf")) |
|  | if(flnm != "") { |
|  | pdf(flnm) |
|  | par(mfrow = c(1, 1)) |
|  | hist(out$qvalues, main = "Histogram of Q-Values", xlab = "") |
|  | dev.off() |
|  | postMsg("Plot saved.\n") |
|  | } |
|  | else |
|  | postMsg("No file selected. Plot not saved.\n") |
|  | } |
|  | } |
|  |  |
|  | else { |
|  | if(is.null(out)) |
|  | postMsg("ERROR: Q-values haven't been computed yet.\n") |
|  | else if(class(out) == "qvalue") { |
|  | flnm <- tclvalue(tkgetSaveFile(defaultextension = "pdf")) |
|  | if(flnm != "") { |
|  | pdf(flnm) |
|  | qplot(out, rng = as.numeric(c(tclvalue(from.var.2), tclvalue(to.var.2)))) |
|  | dev.off() |
|  | postMsg("Plot saved.\n") |
|  | } |
|  | else |
|  | postMsg("No file selected. Plot not saved.\n") |
|  | } |
|  | } |
|  | } |
|  |  |
|  | postMsg <- function(msg) { |
|  | tkconfigure(message.txt, state = "normal") |
|  | tkinsert(message.txt, "end", msg) |
|  | tkconfigure(message.txt, state = "disabled") |
|  | } |
|  |  |
|  | errorHandler <- function() { |
|  | postMsg(paste("An R error has occurred: ", geterrmessage(), sep = "")) |
|  | } |
|  |  |
|  | ## Reroute R errors to the message box |
|  | options(error = errorHandler, show.error.messages = FALSE) |
|  |  |
|  | ############## |
|  | ## GUI code ## |
|  | ############## |
|  |  |
|  | ## Top level |
|  | base <- tktoplevel() |
|  | tkwm.title(base, "QVALUE") |
|  |  |
|  | top.frm <- tkframe(base, borderwidth = 2) |
|  |  |
|  | ## P-Value frame contains text field, browse button, load button, histogram button. |
|  | pValue.frm <- tkframe(top.frm, relief = "raised", bd = 2) |
|  | tkpack(tklabel(pValue.frm, text = "Read P-Values:", font = titleFont), anchor = "w") |
|  | pValueInset.frm <- tkframe(pValue.frm, relief = "groove", bd = 2) |
|  |  |
|  | inFileName.frm <- tkframe(pValueInset.frm) |
|  | inFileName.lbl <- tklabel(inFileName.frm, text = "File Name:", font = normalFont) |
|  | inFileName.ety <- tkentry(inFileName.frm, textvariable = inFileName.var, font = normalFont, |
|  | justify = "center") |
|  | tkpack(inFileName.lbl, side = "left") |
|  | tkpack(inFileName.ety, side = "right", fill = "x", expand = TRUE) |
|  | tkpack(inFileName.frm, fill = "x", expand = TRUE) |
|  |  |
|  | pButtons.frm <- tkframe(pValueInset.frm) |
|  | browse.btn <- tkbutton(pButtons.frm, text = "Browse", font = normalFont, command = findPVals) |
|  | load.btn <- tkbutton(pButtons.frm, text = "Load", font = normalFont, command = readPVals) |
|  | tkgrid(browse.btn, load.btn) |
|  | tkpack(pButtons.frm, anchor = "e") |
|  | tkpack(pValueInset.frm, fill = "x") |
|  |  |
|  | ## Options frame allows user to specify lambda and pi_0 estimation method. |
|  | options.frm <- tkframe(top.frm, relief = "raised", bd = 2) |
|  | tkpack(tklabel(options.frm, text = "Optional Arguments:", font = titleFont), anchor = "w") |
|  | optionsInset.frm <- tkframe(options.frm, relief = "groove", bd = 2) |
|  |  |
|  | #### Specify lambda |
|  | lambdaLabel.frm <- tkframe(optionsInset.frm) |
|  | tkpack(tklabel(lambdaLabel.frm, text = "Specify lambda:", font = normalFont), anchor = "w") |
|  | tkpack(lambdaLabel.frm, fill = "x", expand = TRUE) |
|  |  |
|  | lambdaRange.frm <- tkframe(optionsInset.frm, padx = 10) |
|  | range.rbtn <- tkradiobutton(lambdaRange.frm, text = "Range", font = normalFont, value = 1, |
|  | variable = lambda.var, command = lambda.fnc) |
|  | from.lbl.1 <- tklabel(lambdaRange.frm, text = "from:", font = normalFont) |
|  | to.lbl.1 <- tklabel(lambdaRange.frm, text = "to:", font = normalFont) |
|  | by.lbl.1 <- tklabel(lambdaRange.frm, text = "by:", font = normalFont) |
|  | from.ety.1 <- tkentry(lambdaRange.frm, textvariable = from.var.1, font = normalFont, width = 5, |
|  | justify = "center") |
|  | to.ety.1 <- tkentry(lambdaRange.frm, textvariable = to.var.1, font = normalFont, width = 5, |
|  | justify = "center") |
|  | by.ety.1 <- tkentry(lambdaRange.frm, textvariable = by.var.1, font = normalFont, width = 5, |
|  | justify = "center") |
|  | tkpack(range.rbtn, side = "left", anchor = "w") |
|  | tkpack(from.lbl.1, side = "left") |
|  | tkpack(from.ety.1, side = "left") |
|  | tkpack(to.lbl.1, side = "left") |
|  | tkpack(to.ety.1, side = "left") |
|  | tkpack(by.lbl.1, side = "left") |
|  | tkpack(by.ety.1, side = "left") |
|  | tkpack(lambdaRange.frm, fill = "x", expand = TRUE) |
|  |  |
|  | lambdaSingle.frm <- tkframe(optionsInset.frm, padx = 10) |
|  | single.rbtn <- tkradiobutton(lambdaSingle.frm, text = "Single No.:", font = normalFont, value = 0, |
|  | variable = lambda.var, command = lambda.fnc) |
|  | single.ety <- tkentry(lambdaSingle.frm, textvariable = single.var, font = normalFont, width = 5, |
|  | state = "disabled", justify = "center") |
|  | tkpack(single.rbtn, side = "left", anchor = "w") |
|  | tkpack(single.ety, side = "left") |
|  | tkpack(lambdaSingle.frm, fill = "x", expand = TRUE) |
|  |  |
|  | #### Specify bootstrap or smoother method |
|  | methodLabel.frm <- tkframe(optionsInset.frm) |
|  | tkpack(tklabel(methodLabel.frm, text = "Choose pi_0 method:", font = normalFont), anchor = "w") |
|  | tkpack(methodLabel.frm, fill = "x", expand = TRUE) |
|  |  |
|  | methodSmooth.frm <- tkframe(optionsInset.frm, padx = 10) |
|  | smoother.rbtn <- tkradiobutton(methodSmooth.frm, text = "Smoother", font = normalFont, value = 1, |
|  | variable = pi0.var, command = smoother.fnc) |
|  | smoothOptions.btn <- tkbutton(methodSmooth.frm, text = "Advanced Options", font = normalFont, |
|  | command = smoothOptions.fnc) |
|  | tkpack(smoother.rbtn, side = "left", anchor = "w") |
|  | tkpack(smoothOptions.btn, side = "left") |
|  | tkpack(methodSmooth.frm, fill = "x", expand = TRUE) |
|  |  |
|  | methodBstrap.frm <- tkframe(optionsInset.frm, padx = 10) |
|  | bootstrap.rbtn <- tkradiobutton(methodBstrap.frm, text = "Bootstrap", font = normalFont, value = 0, |
|  | variable = pi0.var, command = smoother.fnc) |
|  | tkpack(bootstrap.rbtn, side = "left", anchor = "w") |
|  | tkpack(methodBstrap.frm, fill = "x", expand = TRUE) |
|  |  |
|  | #### Specify robust method |
|  | robust.frm <- tkframe(optionsInset.frm) |
|  | robust.cbtn <- tkcheckbutton(robust.frm, text = "Use robust method", font = normalFont, |
|  | variable = robust.var) |
|  | tkpack(robust.cbtn, anchor = "w") |
|  | tkpack(robust.frm, fill = "x", expand = TRUE) |
|  |  |
|  | #### Specify FDR level |
|  | level.frm <- tkframe(optionsInset.frm) |
|  | level.cbtn <- tkcheckbutton(level.frm, text = "Specify FDR level:", font = normalFont, |
|  | variable = levelSpec.var, command = level.fnc) |
|  | level.ety <- tkentry(level.frm, textvariable = level.var, font = normalFont, width = 5, |
|  | state = "disabled", justify = "center") |
|  | tkpack(level.cbtn, side = "left", anchor = "w") |
|  | tkpack(level.ety, side = "left") |
|  | tkpack(level.frm, fill = "x", expand = TRUE) |
|  | tkpack(optionsInset.frm, fill = "x", expand = TRUE) |
|  |  |
|  | ## Action frame |
|  | action.frm <- tkframe(top.frm, relief = "raised", bd = 2) |
|  | tkpack(tklabel(action.frm, text = "Compute Q-Values:", font = titleFont), anchor = "w") |
|  | actionInset.frm <- tkframe(action.frm, relief = "groove", bd = 2) |
|  |  |
|  | execute.btn <- tkbutton(actionInset.frm, text = "Execute", font = normalFont, |
|  | command = execute.fnc) |
|  | saveOutput.btn <- tkbutton(actionInset.frm, text = "Save Output", font = normalFont, |
|  | command = saveOutput.fnc) |
|  | tkpack(saveOutput.btn, side = "right", anchor = "e") ## padx argument here spreads buttons out |
|  | tkpack(execute.btn, side = "right") |
|  | tkpack(actionInset.frm, fill = "x", expand = TRUE) |
|  |  |
|  | ## Plot frame |
|  | plot.frm <- tkframe(top.frm, relief = "raised", bd = 2) |
|  | tkpack(tklabel(plot.frm, text = "Plots:", font = titleFont), anchor = "w") |
|  | plotInset.frm <- tkframe(plot.frm, relief = "groove", bd = 2) |
|  |  |
|  | pHist.frm <- tkframe(plotInset.frm, padx = 10) |
|  | pHist.rbtn <- tkradiobutton(pHist.frm, text = "P-value histogram", font = normalFont, value = 1, |
|  | variable = plotChoice.var, command = plotChoice.fnc) |
|  | tkpack(pHist.rbtn, side = "left", anchor = "w") |
|  | tkpack(pHist.frm, fill = "x", expand = TRUE) |
|  |  |
|  | qHist.frm <- tkframe(plotInset.frm, padx = 10) |
|  | qHist.rbtn <- tkradiobutton(qHist.frm, text = "Q-value histogram", font = normalFont, value = 2, |
|  | variable = plotChoice.var, command = plotChoice.fnc) |
|  | tkpack(qHist.rbtn, side = "left", anchor = "w") |
|  | tkpack(qHist.frm, fill = "x", expand = TRUE) |
|  |  |
|  | qPlots.frm <- tkframe(plotInset.frm, padx = 10) |
|  | qPlots.rbtn <- tkradiobutton(qPlots.frm, text = "Q-plots,", font = normalFont, value = 3, |
|  | variable = plotChoice.var, command = plotChoice.fnc) |
|  | from.lbl.2 <- tklabel(qPlots.frm, text = "range from:", font = normalFont) |
|  | to.lbl.2 <- tklabel(qPlots.frm, text = "to:", font = normalFont) |
|  | from.ety.2 <- tkentry(qPlots.frm, textvariable = from.var.2, font = normalFont, width = 7, |
|  | state = "disabled", justify = "center") |
|  | to.ety.2 <- tkentry(qPlots.frm, textvariable = to.var.2, font = normalFont, width = 7, |
|  | state = "disabled", justify = "center") |
|  | tkpack(qPlots.rbtn, side = "left", anchor = "w") |
|  | tkpack(from.lbl.2, side = "left") |
|  | tkpack(from.ety.2, side = "left") |
|  | tkpack(to.lbl.2, side = "left") |
|  | tkpack(to.ety.2, side = "left") |
|  | tkpack(qPlots.frm, fill = "x", expand = TRUE) |
|  |  |
|  | plot.btn <- tkbutton(plotInset.frm, text = "Make Plot", font = normalFont, command = plot.fnc) |
|  | savePlot.btn <- tkbutton(plotInset.frm, text = "Save Plot to PDF", font = normalFont, command = savePlot.fnc) |
|  | tkpack(savePlot.btn, side = "right", anchor = "e") |
|  | tkpack(plot.btn, side = "right") |
|  | tkpack(plotInset.frm, fill = "x", expand = TRUE) |
|  |  |
|  | ## Message box |
|  | message.frm <- tkframe(top.frm, relief = "raised", bd = 2) |
|  | message.txt <- tktext(message.frm, bg = "white", font = normalFont, height = 5, width = 5) |
|  | message.scr <- tkscrollbar(message.frm, command = function(...) tkyview(message.txt, ...)) |
|  | tkconfigure(message.txt, yscrollcommand = function(...) tkset(message.scr, ...)) |
|  | tkpack(message.txt, side = "left", fill = "x", expand = TRUE) |
|  | tkpack(message.scr, side = "right", fill = "y") |
|  |  |
|  | tkpack(pValue.frm, fill = "x") |
|  | tkpack(options.frm, fill = "x") |
|  | tkpack(action.frm, fill = "x") |
|  | tkpack(plot.frm, fill = "x") |
|  | tkpack(message.frm, fill = "x") |
|  | tkpack(top.frm) |
|  |  |
|  | tkwm.focusmodel(base, "active") |
|  |  |
|  | } |
|  |  |
|  | } |

[qvalue.R](https://github.com/Neal050617/RFCV/commit/d33765970435dca44d97a17955a4a4845763de54#diff-672bcabf639f615b163b20bf66839534)

**[View file](https://github.com/Neal050617/RFCV/blob/d33765970435dca44d97a17955a4a4845763de54/random_forest4key_out_select.pl)**

**82** [random_forest4key_out_select.pl](https://github.com/Neal050617/RFCV/commit/d33765970435dca44d97a17955a4a4845763de54#diff-565d02e78b39fe6aca7d05fe648c1c01)

| @@ -0,0 +1,82 @@ | |
| --- | --- |
|  | #! /usr/bin/perl |
|  | if(@ARGV<4){ |
|  | print "random_forest4key_out_select.pl biom otu_table group threshold\n"; |
|  | exit; |
|  | } |
|  | my $biom = shift; |
|  | my $otu_table = shift; |
|  | my $group = shift; |
|  | my $threshold = shift; #0.005\|\|0.003; |
|  | #print "$threshold\n"; |
|  | my @group_array; |
|  | my %groups; |
|  | my %group_details; |
|  | my %group_details2; |
|  | my $last =""; |
|  | open(GROUP,"<$group") or die; |
|  | my $head = <GROUP>; |
|  | chomp $head; |
|  | while(<GROUP>){ |
|  | chomp $_; |
|  | my @line = split /\t/,$_; |
|  | if($line[1] ne $last){ |
|  | $group_array[@group_array] = $line[1]; |
|  | } |
|  | $last = $line[1]; |
|  | $groups{$line[0]} = $line[1]; |
|  | if(exists $group_details{$line[1]}){ |
|  | $group_details{$line[1]} .= ";".$line[0]; |
|  | $group_details2{$line[1]} .= ";".$line[0]."\t".$line[1]; |
|  | }else{ |
|  | $group_details{$line[1]} .= $line[0]; |
|  | $group_details2{$line[1]} .= $line[0]."\t".$line[1]; |
|  | } |
|  | } |
|  | close(GROUP); |
|  |  |
|  | open(CMD,">random_forest.sh") or die; my @dirs; |
|  | for($i=0;$i<@group_array;$i++){ |
|  | for($j=$i+1;$j<@group_array;$j++){ |
|  | #print "$group_array[$i]\t$group_array[$j]\n"; |
|  | my $list = "$group_array[$i]-$group_array[$j].samples.list"; |
|  | my $map = "map.$group_array[$i]-$group_array[$j].txt"; |
|  | my $dir = "$group_array[$i]-$group_array[$j]"; |
|  | $dirs[@dirs] = $dir; |
|  | open(LIST,">$list") or die; |
|  | open(MAP,">$map") or die; |
|  | $group_details{$group_array[$i]} =~ s/;/**\n**/g; |
|  | $group_details{$group_array[$j]} =~ s/;/**\n**/g; |
|  | print LIST "$group_details{$group_array[$i]}\n$group_details{$group_array[$j]}\n"; |
|  | close(LIST); |
|  |  |
|  | print MAP "#sample\tgroup\n"; |
|  | $group_details2{$group_array[$i]} =~ s/;/**\n**/g; |
|  | $group_details2{$group_array[$j]} =~ s/;/**\n**/g; |
|  | print MAP "$group_details2{$group_array[$i]}\n$group_details2{$group_array[$j]}\n"; |
|  | close(MAP); |
|  | my $biom_tmp = "otu_table_"."$group_array[$i]-$group_array[$j]".".biom"; |
|  | print CMD "filter_samples_from_otu_table.py -i $biom --sample_id_fp $list -o $biom_tmp\n"; |
|  | print CMD "supervised_learning.py -i $biom_tmp -m $map -c group -o $dir --ntree 1000\n"; |
|  | #print CMD "cat */feature_importance_scores.txt > feature_importance_scores-all.txt\n"; |
|  |  |
|  | } |
|  | } |
|  | print CMD "cat */feature_importance_scores.txt > feature_importance_scores-all.txt\n"; |
|  | print CMD "more feature_importance_scores-all.txt \|awk \'\$2>$threshold"."{print \$1}\'\|sort \|uniq \|grep OTU >OTU-extract.all.list\n"; |
|  | print CMD "less $otu_table \|head -1 >OTU-extract.all.xls\n"; |
|  | print CMD "less $otu_table \|grep -wf OTU-extract.all.list >>OTU-extract.all.xls\n"; |
|  | close(CMD); |
|  | print "group vs group\tOTU_extract\n"; |
|  | `sh random_forest.sh`; |
|  | for (my $i=0;$i<@dirs;$i++){ |
|  | my $ccc = "more $dirs[$i]/feature_importance_scores.txt \|awk \'\$2>$threshold"."{print \$1}\'"; |
|  | #print "more $dirs[$i]/feature_importance_scores.txt \|awk \'\$2>$threshold"."{print \$1}\'\|wc -l\n"; |
|  | my $tmp = `$ccc\|wc -l`; |
|  | chomp $tmp; |
|  | $tmp--; |
|  | print "$dirs[$i]\t$tmp\n"; |
|  | } |
|  | my $tmp = `wc -l OTU-extract.all.xls`; |
|  | chomp $tmp; |
|  | $tmp--; |
|  | print "union_all\t$tmp\n"; |

**[View file](https://github.com/Neal050617/RFCV/blob/d33765970435dca44d97a17955a4a4845763de54/wilcox.py)**

**81** [wilcox.py](https://github.com/Neal050617/RFCV/commit/d33765970435dca44d97a17955a4a4845763de54#diff-f1a39accf4cf229e94e8ded3b9ac46f9)

|  |  | @@ -0,0 +1,81 @@ | |
| --- | --- | --- | --- |
|  |  |  | #!/usr/bin/env python |
|  |  |  | # last updata 20150519 by yuguo |
|  |  |  | import argparse |
|  |  |  | import os |
|  |  |  |  |
|  |  |  | def option(): |
|  |  |  | par= argparse.ArgumentParser() |
|  |  |  | par.add_argument("-i",metavar='[infiles]',required=True, help='Input abundance profile file') |
|  |  |  | par.add_argument("-o",metavar='[ouput]',help="Output file name.",required=True) |
|  |  |  | par.add_argument("-g",metavar='[group file]',help="goup file with two columns ,first clo are samples,second indicates group label.",required=True) |
|  |  |  | par.add_argument("-c",metavar='[c1-c2]',help="two group label to compare,sep with '-'. ",required=True) |
|  |  |  | args = par.parse_args() |
|  |  |  | return args |
|  |  |  |  |
|  |  |  | def wilcox(infile,outfile,gfile,c1,c2): |
|  |  |  | cmd=''' |
|  |  |  | #library(qvalue) |
|  |  |  | source(\"/work/scripts/16s/qvalue.R\") |
|  |  |  | data=read.table("'''+infile+'''",sep="\\t") |
|  |  |  | #samp <-as.vector(data[1,-1]) |
|  |  |  | samp <-t(data[1,-1]) |
|  |  |  | head=as.character(data[1,1]) |
|  |  |  | data <-data[-1,] |
|  |  |  | rownames(data)<-data[,1] |
|  |  |  | data <-data[,-1] |
|  |  |  | colnames(data)<-samp |
|  |  |  |  |
|  |  |  | group=read.table("'''+gfile+'''",sep="\\t") |
|  |  |  | #group <-unlist(gat[1,-1]) |
|  |  |  |  |
|  |  |  | g1="'''+c1+'''" |
|  |  |  | g2="'''+c2+'''" |
|  |  |  | gsamp=group[which(group[,2] %in% c(g1,g2)),1] |
|  |  |  | #gsamp1=as.vector(group[which(group[,2] %in% g1),1]) |
|  |  |  | #gsamp2=as.vector(group[which(group[,2] %in% g2),1]) |
|  |  |  | gsamp1=group[which(group[,2] %in% g1),1] |
|  |  |  | gsamp2=group[which(group[,2] %in% g2),1] |
|  |  |  | data <-data[,which(samp %in% gsamp)] |
|  |  |  | samp <-samp[which(samp %in% gsamp)] |
|  |  |  |  |
|  |  |  | data <-data[apply(data,1,function(x)any(x>0)),] |
|  |  |  | #group <-group[which(group %in% c(g1,g2))] |
|  |  |  | #print(colnames(data)) |
|  |  |  | da=data |
|  |  |  | data <-apply(da,2,function(x) as.numeric(x)/sum(as.numeric(x))) |
|  |  |  | rownames(data)<-rownames(da) |
|  |  |  |  |
|  |  |  | out<-matrix(nrow=nrow(data),ncol=6) |
|  |  |  | for(i in 1:nrow(data)){ |
|  |  |  | d1=as.numeric(as.vector(unlist(data[i,which(samp %in% gsamp1)]))) |
|  |  |  | d2=as.numeric(as.vector(unlist(data[i,which(samp %in% gsamp2)]))) |
|  |  |  | wt <-wilcox.test(d1,d2,exact=F) |
|  |  |  | me1 <-mean(d1) |
|  |  |  | me2 <-mean(d2) |
|  |  |  | sd1 <-sd(d1) |
|  |  |  | sd2 <-sd(d2) |
|  |  |  | out[i,]=c(rownames(data)[i],me1,sd1,me2,sd2,wt$p.value) |
|  |  |  | #out[i,1]=rownames(data)[i] |
|  |  |  | #out[i,2]=wt$p.value |
|  |  |  | } |
|  |  |  | qv=qvalue(as.numeric(out[,6]),lambda=0.5) |
|  |  |  | out <-cbind(out,qv$qvalues) |
|  |  |  | colnames(out)=c(" ",paste("mean(",g1,")",sep=''),paste("sd(",g1,")",sep=''),paste("mean(",g2,")",sep=''),paste("sd(",g2,")",sep=''),"p-value","q-value") |
|  |  |  | out_order=out[order(out[,6]),] |
|  |  |  | write.table(out_order,"'''+outfile+'''",sep="\\t",col.names=T,row.names=F) |
|  |  |  | out_choosed=out_order[which(out_order[,6]<0.05 & out_order[,7]<0.01),] |
|  |  |  | data_choosed=data[out_choosed[,1],] |
|  |  |  | data_choosed=cbind(rownames(data_choosed),data_choosed) |
|  |  |  | colnames(data_choosed)[1]=head |
|  |  |  | write.table(data_choosed,paste("'''+outfile+'''",".filtered.xls",sep=""),sep="\t",col.names=T,row.names=F) |
|  |  |  | ''' |
|  |  |  | #print(cmd) |
|  |  |  | cmdfile=open("tmp.r",'w') |
|  |  |  | cmdfile.write(cmd) |
|  |  |  | cmdfile.close() |
|  |  |  | os.system("Rscript tmp.r") |
|  |  |  |  |
|  |  |  | if __name__ == "__main__": |
|  |  |  | ### get options |
|  |  |  | opts=option() |
|  |  |  | wilcox(opts.i,opts.o,opts.g,opts.c.split('-')[0],opts.c.split('-')[1]) |
